# *Streptococcus mutans* PrsA mediates AtlA secretion contributing to extracellular DNA release and biofilm formation in the pathogenesis of infective endocarditis

**DOI:** 10.1080/21505594.2022.2105351

**Published:** 2022-08-11

**Authors:** Chih-Chieh Hsu, Ron-Bin Hsu, Xoong-Harng Oon, Ya-Tang Chen, Jeng-Wei Chen, Che-Hao Hsu, Yu-Min Kuo, Yi-Hsien Shih, Jean-San Chia, Chiau-Jing Jung

**Affiliations:** a Graduate Institute of Clinical Dentistry, School of Dentistry, National Taiwan University, Taipei, Taiwan; b Graduate Institute of Oral Biology, School of Dentistry, National Taiwan University, Taipei, Taiwan; c Department of Surgery, Division of Cardiovascular Surgery, National Taiwan University Hospital , College of Medicine, National Taiwan University, Taipei, Taiwan; d Graduate Institute of Medical Sciences, College of Medicine, Taipei medical University, Taipei, Taiwan; e Department of Microbiology and Immunology, School of Medicine, College of Medicine, Taipei Medical University, Taipei, Taiwan; f Graduate Institute of Clinical Medicine, College of Medicine, National Taiwan University, Taipei, Taiwan; g Division of Allergy, Immunology, and Rheumatology, Department of Internal Medicine, National Taiwan University Hospital, Taipei, Taiwan; h Department of Dermatology, Taipei Medical University-Shuang Ho Hospital, New Taipei City, Taiwan; i Department of Dermatology, School of Medicine, College of Medicine, Taipei Medical University, Taipei, Taiwan

**Keywords:** *Streptococcus mutans*, infective endocarditis, PrsA, AtlA, extracellular DNA

## Abstract

The role of secretion chaperone-regulated virulence proteins in the pathogenesis of infective endocarditis (IE) induced by viridans streptococci such as *Streptococcus mutans* is unclear. In this study, we investigated the contribution of the foldase protein PrsA, a putative parvulin-type peptidyl-prolyl isomerase, to the pathogenesis of *S. mutans*-induced IE. We found that a *prsA*-deficient strain had reduced virulence in terms of formation of vegetation on damaged heart valves, as well as reduced autolysis activity, eDNA release and biofilm formation capacity. The secretion and surface exposure of AtlA *in vitro* was reduced in the *prsA*-deficient mutant strain, and complementation of recombinant AtlA in the culture medium restored a wild type biofilm phenotype of the *prsA*-deficient mutant strain. This result suggests that secretion and surface localization of AtlA is regulated by PrsA during biofilm formation. Together, these results demonstrate that *S. mutans* PrsA could regulate AtlA-mediated eDNA release to contribute to biofilm formation in the pathogenesis of IE.

## Introduction

Bacterial biofilm formation is a determining virulence factor for bacteria that cause infectious disease [1]. Biofilms not only mediate bacterial colonization but also contribute to bacterial resistance to antibiotic treatment [2,3]. Infective endocarditis (IE) is a typical infectious disease related to formation of biofilms that are frequently induced by staphylococci or oral streptococci [1,4]. In contrast to formation of biofilms on polystyrene surfaces *in vitro* that mainly comprise a carbohydrate matrix, our previous studies demonstrated that host factors, platelets, and neutrophil extracellular traps (NETs) contribute to bacteria biofilm formation and vegetation maturation in the pathogenesis of IE [5,6]. In addition, bacterial extracellular DNA (eDNA) plays a crucial role in *Streptococcus mutans* biofilm formation inside vegetation on affected heart valves [7,8].

Bacterial eDNA release to biofilm matrices is mainly mediated by two specific mechanisms: autolysis and vesicle-associated active secretion [9]. Bacterial autolysin mediates bacterial lysis with consequent release of eDNA into biofilms formed by diverse species [10,11]. Our previous studies demonstrated that AtlA, an *S. mutans* autolysin, mediates bacterial eDNA release, which contributes to bacterial biofilm formation on heart valves in IE [7]. AtlA does not contain the typical LPXTG cell wall anchor domain [12]. As such, the mechanism of AtlA surface localization is unclear. A recent study indicated that AtlA specifically binds to immature serotype c carbohydrates in the cell wall and mediates bacterial cell wall separation [13]. These data implied that AtlA can be released from the bacteria and bind to bacterial cell walls to mediate bacterial autolysis and eDNA release.

PrsA is a putative parvulin-type peptidyl-prolyl isomerase (PPIase) chaperone of *S. mutans* [14]. PrsA homologs are present in diverse pathogens, including *Listeria monocytogenes* and *Staphylococcus aureus*, and are demonstrated to play important roles in the secretion and surface localization of virulence proteins to contribute to bacterial virulence and infection [15–17]. Studies conducted on pathogenic streptococci also showed that PrsA regulates bacterial pathogenicity by modulating secretion of virulence factors [18,19]. In *S. mutans*, a *prsA*-mutant strain showed a marked increase in chain length, and reduced ability to produce the insoluble glucan and mutacin IV. Deletion of *prsA* also affects the surface localization of proteins, including glucosyltransferases and SpaP, and influenced the ability to form sucrose-dependent biofilms with aberrant architecture [14,20]. These findings provided information about the contribution of PrsA *S. mutans* virulence in the pathogenesis of dental caries, but whether PrsA mediates secretion or surface localization of bacterial virulence factors involved in IE pathogenesis is unclear.

In this study, we investigated the role of PrsA in IE pathogenesis in an animal model of endocarditis in rats. We showed that PrsA mediates secretion and surface localization of AtlA, which contributes to bacterial eDNA release and biofilm formation in *S. mutans*-induced endocarditis.

## Materials and methods

### Bacterial strains and plasmids

*S.mutans* GS5 wild-type, *prsA*-deficient (Δ*prsA*), and *prsA*-complemented (comΔ*prsA*) strains were grown in BHI broth (Difco Laboratories Inc., Detroit, MI, USA). A shuttle plasmid containing the *GFPuv* sequence (pPDGFPuv) was used to generate green fluorescent protein (GFP)-tagged bacteria as previously described [21]. Spectinomycin (500 μg/mL; pPDGFPuv), kanamycin (500 μg/mL; Δ*prsA* and comΔ*prsA*), and chloramphenicol (5 μg/mL; comΔ*prsA*) were used to select the colonies.

## Construction of δprsa and comδprsa strains

To generate the deletion mutant strain of *prsA*, a ligation-PCR mutagenesis strategy with a promoterless kanamycin cassette was used as previously described [22]. The upstream and downstream sequences of *prsA* and the promoterless kanamycin resistance gene fragment were amplified from *S. mutans* GS5 genomic DNA and the pALH124 plasmid, respectively, using the primers listed in Table 1 [23]. The primers were designed according to the *S. mutans* GS5 genome database (https://www.ncbi.nlm.nih.gov/genome/). PCR reaction products were digested with *Eco*RI and ligated with T4 ligase. The ligation was directly transformed into wild-type *S. mutans* GS5 or a clinical isolate, 4152, and the correct deletion mutant strain was selected by PCR amplification. The loss of *prsA* expression and that of genes downstream of *prsA* was confirmed by RT-PCR. For construction of the *prsA*-complemented strain (comΔ*prsA*), the *prsA* promoter and *prsA* gene fragments were PCR-amplified using the primers listed in Table 1. The PCR products of the promoter and *prsA* gene were digested with *Xba*I and cloned into the pMC340B_Cm plasmid [21]. The complementation plasmid was transformed into the Δ*prsA* strain to generate the *prsA-*complemented strain (comΔ*prsA*). Complementation of *prsA* was screened by PCR amplification, and further confirmed by RT-PCR and western blotting.

**Table 1.** PCR primers used in the current study.

Primers	Sequence (5‘to 3’) (restriction sites: underlined)	Target
**For generation of deletion mutants and the complementation strains**		
prsA_BF	TGAGAATGCTCCACAAGC	upstream fragment of *prsA*
prsA_BR_EcoRI	CGGAATTCGCCGCTAAAGTCACAATAG	
prsA_AF_EcoRI	CGGAATTCAGAGACAACAGCAGCAGA	downstream fragment of *prsA*
prsA_AR	AGGAAGGAAGGTCCAAGT	
KanF		kanamycin resistance gene fragment
KanR		
prsAPF_SphI	GTACGAGCATGCTGATATGACTGAGTCGACC	*prsA* operon promoter
prsAPR_XbaI	TACGTCTCTAGATGTTTGTGGCATATTTTCTC	
prsAF_XbaI	CTAGTATCTAGAATGAAAAAACGTACGATTGC	*prsA*
prsAR_XbaI	CGTGATTCTAGACAACATTCGTCAATCTTTAC	
**Semiquantification of eDNA**		
16S-F	AGAGTTTGATCMTGGCTCAG	16S rRNA gene
16S-R	GGTTACCTTGTTACGACTT	

## A rat model of S. mutans-induced IE

A modified rat model of experimental streptococcal endocarditis was used to investigate the IE pathogenesis as previously described [24]. The conduct of animal experiments was approved by the Institutional Animal Care and Use Committee (National Taiwan University, Taipei, Taiwan). Briefly, an injury in the aortic valves was created by the insertion of a polyethylene tube with a stainless-steel wire embedded inside into the left carotid artery. Twenty-four hours later, rats were intravenously infected with 1 × 10^9^ CFU of bacteria. At 24 h post-infection, the vegetation was harvested and weighed. To quantify bacterial colonization, the vegetation samples were homogenized by ultrasonication. The vegetation homogenates from six rats from each group were plated and the resulting colonies were counted. For the observation of biofilms by confocal laser scanning microscopy (CLSM; Leica TCS SP5), *S. mutans* GS5 wild-type and the mutant strains were transformed with pPDGFPuv [21]. *S. mutans* (1 × 10^9^ CFU) were intravenously infected into the tail veins of catheterized rats and the vegetation was harvested 24 h later. For each vegetation sample, three regions were evaluated by confocal microscopy, and a representative image is shown. To detect bacterial eDNA, the vegetation was stained with propidium iodide (PI) and the bacterial biofilms were observed by confocal microscopy (Leica TCS SP5).

## Biofilm formation assays

The eDNA-dependent biofilm formation assay was performed as previously described [7]. Briefly, the bacterial biofilms were grown by inoculating approximately 10^7^ CFU of *S. mutans* in 200 μL defined M4 medium with or without the indicated concentration of *S. mutans* chromosome DNA or recombinant AtlA in a 96-well polystyrene microtiter plate. *S. mutans* chromosome DNA was extracted using a commercial kit (Geno Plus Genomic DNA Extraction kit; Viogene, Taipei, Taiwan), and the recombinant AtlA was purified using nickel chelating chromatography as previously described [21]. For quantification, the biofilms were stained with 0.1% crystal violet dye, which was extracted using extraction buffer (20% methanol and 80% acetone). The absorbance of the extracted crystal violet dye (550 nM) was measured with a MicroELISA reader (Dynatech Corp., Alexandria, VA, USA). All the biofilm experiments were performed in triplicate and repeated at least three times. For the observation by CLSM, GS5 wild-type and mutant strains were transformed with pPDGFPuv and cultured in 24-well plates for 16 h at 37 °C. After gently washing three times with PBS, the biofilms that formed on the glass coverslip were fixed with 2% paraformaldehyde for 30 min and then stained with 10 μM PI for 10 min before observation with CLSM. Three different regions were evaluated for each biofilm sample and a representative region is shown.

## Autolysis assay

Autolysis was performed as previously described. Bacterial cells in the exponential growth phase (OD_550_ = 0.9) were harvested by centrifugation. After washing three times with PBS, bacterial cells were resuspended in autolysis buffer [25] with or without recombinant AtlA to OD_550_ = 0.9. Bacterial autolysis was monitored by measuring the OD_550_ of the cell suspension. All experiments were performed in triplicate and repeated at least three times.

## Semiquantification of eDNA

Bacterial eDNA released into the culture medium was detected as previously described [7]. Briefly, culture tubes of wild-type and isogenic mutant strains were agitated using a vortex mixer (Vortex Genie, Scientific Industries, Inc.) at 3,200 rpm for 30 sec to disrupt the biofilm, and the culture supernatants were harvested by centrifugation, followed by filtration using filters with 0.45 μm pore size to remove bacteria cells. The eDNA in the supernatant was semi-quantified by conventional PCR amplification (25 cycles) or quantified PCR (qPCR) analysis using specific bacterial 16S rRNA primers listed in Table 1. Culture medium containing *S. mutans* GS5 genomic DNA or without seeding bacteria was used as a positive and negative control, respectively. The experiments were performed in triplicate and repeated at least three times. For detecting bacterial eDNA inside the vegetation, a Gentra Puregene Tissue kit (Qiagen, Hilden, Germany) was used to extract the total DNA without lysing the bacteria [7]. The extracted bacterial eDNA was then semi-quantified by PCR amplification (25 cycles) or qPCR analysis using specific bacterial 16S rRNA primers. Three rats were used for detection of bacterial eDNA, and each rat represented one independent experiment.

## Immunofluorescence staining of AtlA exposure on bacterial surface

Briefly, the bacteria were fixed with 4% paraformaldehyde for 30 min and blocked with 3% bovine serum albumin in PBS for 1 hour. Samples were then incubated with a rabbit anti-AtlA antibody (1:200 dilution) for 2 hours [21], followed by Texas red-conjugated anti-rabbit secondary antibodies (1:400 dilution) for 2 hours. AtlA exposure on bacterial surface was observed using Apotome 3D Super Resolution Microscopy (ZEISS, Oberkochen, Germany). Three different regions were evaluated for each sample and a representative region is shown.

## Cell wall pulldown assay

Binding of AtlA in the culture medium to bacterial cell walls was detected by a cell wall pulldown assay that was performed as previously described with some modification [13]. Briefly, the supernatant of overnight bacterial cultures was harvested by centrifugation and incubated with lyophilized cell walls on a rotator for 2 h. The lyophilized cell walls were prepared as previously described with modifications [26]. Briefly, the exponential growth phase cells (OD550 = 0.9) were collected by centrifugation. The cell pellet was washed three times with iced PBS and resuspended in 5% SDS solution. After boiling for 30 minutes, the bacterial cells were broken with glass beads in a homogenizer (FastPrep FP120 homogenizer; Qbiogene Inc., Montreal, Canada). After the addition of DNase, RNase, trypsin, and protease K, the mixtures were incubated at 37°C for 2 hours. The cell wall fragments were washed with acetone and lyophilized. After the incubation with culture supernatant, the cell walls were collected by centrifugation, and washed three times with PBS buffer. After washing, the cell wall-binding proteins were dissolved in SDS sample buffer and analysed by western blotting using anti-AtlA antibodies [21]. The experiment was performed three times and a representative result is shown.

## Statistical analysis

For differences between more than two groups, a 1-way analysis of variance (ANOVA) with subsequent Bonferroni multiple-comparisons test was used, and a Kruskal–Wallis test followed by Dunn’s test was used for nonparametrically distributed data. Differences were considered to be statistically significant at *P* < 0.05.

## Results

### PrsA-Deficient mutant has reduced formation of vegetation and biofilm in vivo

**Figure 1.**
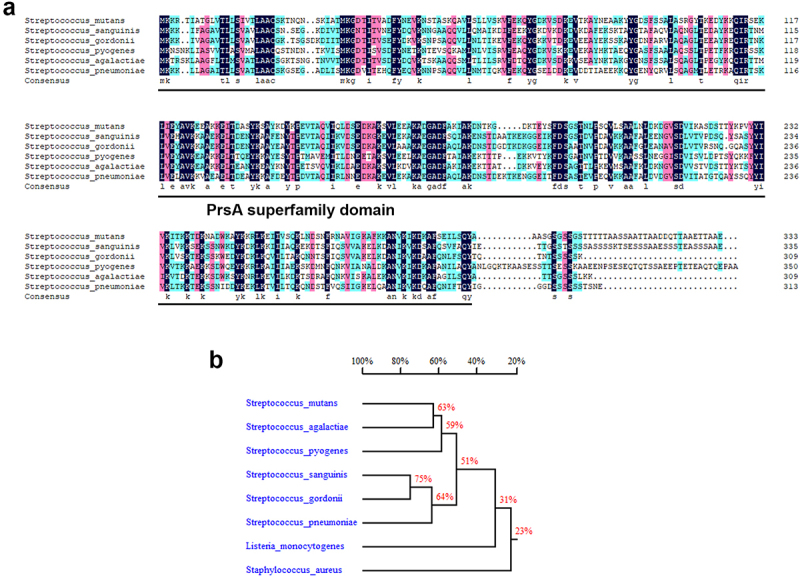
PrsA amino acid sequence conservation among pathogenic streptococci. (a) Alignment of the PrsA amino acid sequences from pathogenic streptococci. The sequences were obtained from the NCBI database. A high degree of sequence conservation was observed. (b) Homology tree of PrsA amino acid sequences from pathogenic streptococci.

**Figure 2.**
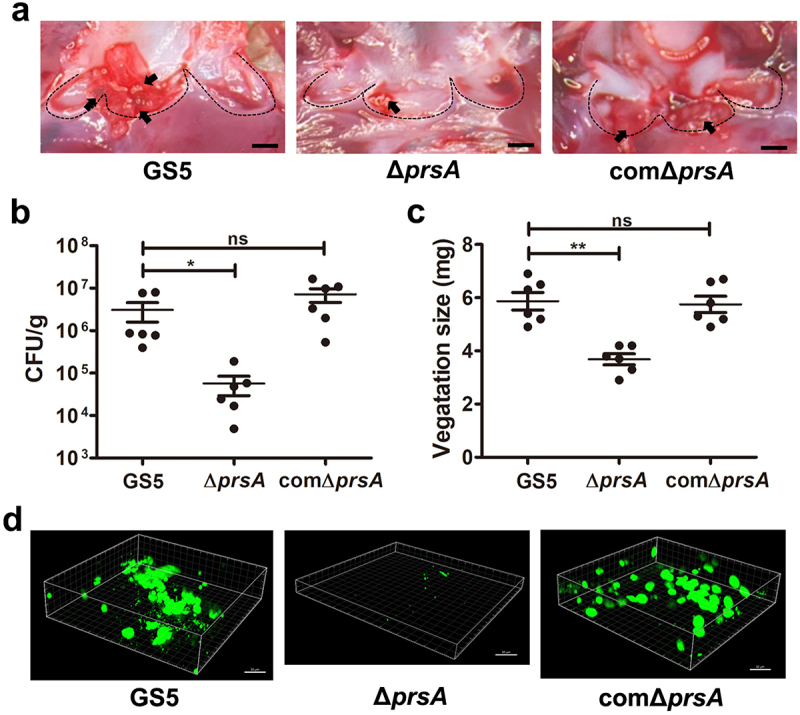
*PrsA*-Deficient mutant strain shows reduced ability to promote vegetation formation in a rat model of IE in rats. Investigation of the role of PrsA in the pathogenesis of IE using GS5 wild-type, Δ*prsA*, and comΔ*prsA* strains in rat models of endocarditis. (a) Vegetation formation on the heart valves of endocarditis rats. Markers represent vegetation (black arrows) and valves (dashed lines). Scale bars represent 1 mm. (b and c) the number of colonized bacteria inside vegetation (b) and vegetation size (c) was measured. Data are presented as a scatter plot with mean ± standard error of the mean. **P < 0.001, *P < 0.05 by Kruskal–Wallis test with subsequent Dunn’s test; ns, not significant. (d) Three-dimensional structure of GFP-tagged *S. mutans* biofilms inside the vegetation that was harvested from injured heart valves and observed by confocal laser scanning microscopy (630× magnification). Bars indicate 50 μm.

PPIase chaperones, including PrsA, mediate secretion of bacterial virulence proteins to contribute to bacterial pathogenesis, including streptococci [15]. Amino acid sequence alignment of PrsA from pathogenic streptococci shows good conservation among PrsA sequences (Figure 1), suggesting that the role of PsrA is similar in streptococcal pathogenesis. To investigate the role PrsA in the pathogenesis of oral streptococci-induced IE, experimental IE rats were intravenously infected with wild-type or *prsA*-deficient (Δ*prsA*) mutant strains of *S. mutans* GS5, or a clinical blood isolate, 4152 or its isogenic *prsA* mutant. The vegetations on the heart valve were harvested 24 hr after infection. The *PrsA*-deficient mutant (Δ*prsA*) exhibited a significantly reduced ability to colonize the heart valve and form vegetation compared with the parental strain (Figure 2(a-c), and Supplementary Fig. S1a-c). Complementation of the *prsA* gene into *prsA*-deficient strains (comΔ*prsA*) restored the ability to form vegetation, thus confirming the role of PrsA (Figure 2(a-c), and Supplementary Fig. S1a-c). To further investigate the role of PrsA in bacterial biofilm formation on heart valves *in vivo*, GFP-tagged wild-type, *prsA*-deficient mutant, and *prsA*-complemented strains were used. The *prsA*-deficient strain consistently showed reduced ability to form biofilms on heart valves compared with the wild-type and complementation strains (Figure 2d and Supplementary Fig. S1d). These data suggested that PrsA plays a role in modulating the ability of bacteria to form biofilm and vegetation on heart valves *in vivo*.

### PrsA mediates release of bacterial eDNA that contributes to bacterial biofilm formation in vitro and in vivo

**Figure 3.**
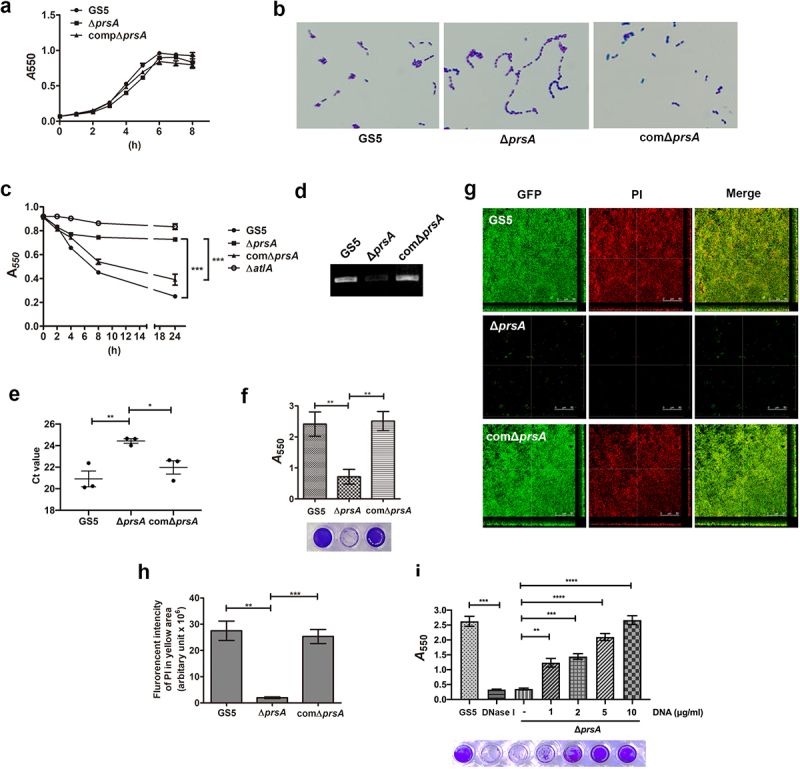
PrsA mediates *S. mutans* cell separation, autolysis, eDNA release, and eDNA-dependent biofilm formation. (a) the bacterial growth was measured by detecting the absorbance of cultures at 550 nm. (b) Appearance of *S. mutans* GS5 wild-type, Δ*prsA*, and comΔ*prsA* in the stationary phase were observed by light microscopy. (c) Bacterial autolysis assessed by measuring the OD_550_ of the cell suspensions. Data are expressed as the mean ± standard deviation of triplicate data. The values at 24 h were analyzed by 1-way ANOVA, ***P < 0.001. (d and e) Semi-quantitative analysis of bacterial eDNA release by conventional PCR and quantitative PCR (qPCR). Conventional PCR products obtained with 16S rRNA primers were analyzed on 1% agarose gels, and data of qPCR are represented as the mean of Ct values with SEM, and analyzed by 1-way ANOVA, *P < 0.001, *P < 0.05. Each spot represents the value from an independent culture. (f) Quantification of *S. mutans* biofilm formation using a crystal violet staining assay. Means of OD_550_ absorbance readings ± standard deviation of triplicate data are shown, and the data were statistically analyzed by 1-way ANOVA. **P < 0.01, ns, not significant. (g) Confocal laser scanning microscopy images of *S. mutans* biofilms (630× magnification). *S. mutans* GS5 wild-type and mutant strains were transformed with pPdgfpuv (green), and bacterial eDNA was stained with 10 μM propidium iodide (PI). GFP, green fluorescent protein. (h) Quantification of eDNA inside biofilms by detecting the fluorescence intensity of propidium iodide staining. The quantified values of the extended focus images of biofilms were determined using ImageJ software. The data were analyzed by 1-way ANOVA from three independent experiments and are presented as the mean value ± standard deviation. **P < 0.01, ***P < 0.001 by. ns, not significant. (i) Quantification of biofilm by crystal violet staining. *S. mutans* GS5 wild-type or Δ*prsA* were grown in culture medium with or without a concentration series of bacterial DNA. The data are presented as the mean ± standard deviation, and were analyzed by 1-way ANOVA. **P < 0.01, ***P < 0.001. The results for a representative experiment from three independent experiments are shown. The results of two other repeats were available in Supplemental Materials.

*In vitro*, the *prsA*-deficient mutant showed a similar growth rate to wild type, but had increased cell chain length (Figure 3(a,b) and Supplementary Fig. S2a and S2b) that is similar to the *atlA*-deficient mutant strain [25]. Our previous study indicated that *S. mutans* autolysin AtlA mediates autolysis and eDNA release to contribute to biofilm formation on the heart valves [7]. Thus, we investigated the role of PrsA in eDNA-dependent biofilm formation. The *prsA*-deficient mutant showed reduced autolysis activity and reduced ability to release eDNA into the culture medium (Figure 3(c-e) and Supplementary Fig. S2c and S2d). Moreover, the ability of the *prsA*-deficient mutant to release eDNA into the biofilm matrix and capacity for eDNA-dependent biofilm formation was diminished (Figure 3(F-h) and Supplementary Fig. S2e-g). Addition of bacterial DNA to the culture medium of the *prsA*-deficient strain restored the bacterial biofilm formation capacity in a dose-dependent manner (Figure 3i and Supplementary Fig. S2 h). These data confirmed the role of eDNA in PrsA-mediated biofilm formation.

**Figure 4.**
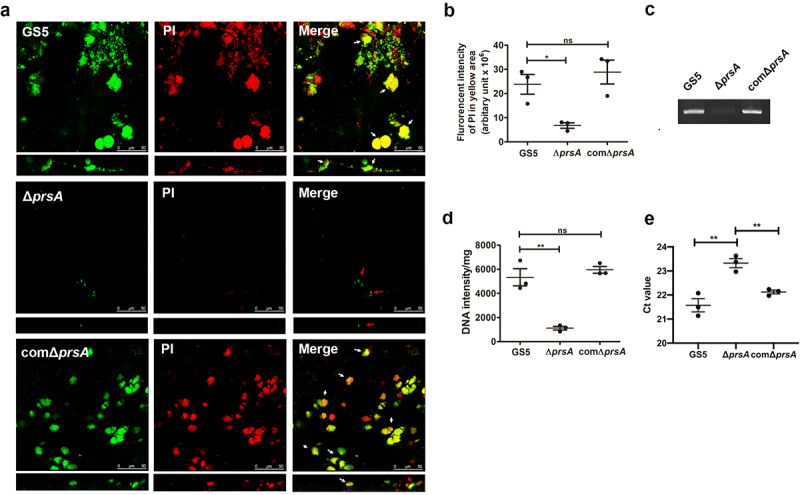
PrsA mediates *in vivo* eDNA-dependent biofilm formation. (a) Confocal laser scanning microscopic images of biofilm formation inside the vegetation (630× magnification). *S. mutans* GS5 wild-type and mutant strains were transformed with pPdgfpuv (green), and bacterial eDNA was stained with 10 μM propidium iodide (PI). GFP, green fluorescent protein. The red arrows indicate small bacterial aggregates without eDNA inside, and white arrows indicate the presence of both *S. mutans* and eDNA (yellow areas). The images shown are representative of three independent experiments. (b) Quantification of the eDNA inside the bacterial biofilms (yellow areas) by measuring the fluorescence intensity of propidium iodide staining. The quantified values of the extended focus images of biofilms were detected using ImageJ software. The data are presented as scatter plots with mean ± standard deviation. ***P < 0.001 by 1-way ANOVA. (c, d and e) Semi-quantification of bacterial eDNA inside the vegetation. Total DNA of vegetation was extracted without lysing bacteria, and the bacterial 16S rRNA gene was amplified by conventional PCR or quantitative PCR (qPCR) with specific primers. Conventional PCR products were analyzed on 1% agarose gels (c) and the intensity was semi-quantified using ImageJ software (d). Data of qPCR are represented as Ct values (e). These data were statistically analyzed by 1-way ANOVA and presented as scatter plots with means ± standard deviation from three independent experiments, ***P < 0.001, **P < 0.01, *P < 0.05.

We next stained vegetation from samples from IE model rats with propidium iodide (PI) to observe bacterial eDNA release or bacterial lysis *in situ* by confocal microscopy (Figure 4a and Supplementary Fig. S3a) [7]. Colocalization of bacteria and DNA was observed (Figure 4a, and Supplementary Fig. S3a, yellow areas indicated by white arrows) for wild-type and *prsA*-complemented strains, suggesting that DNA was embedded inside the bacterial aggregates. Meanwhile, the *prsA*-deficient mutant had only small aggregates that lacked DNA (Figure 4a, and Supplementary Fig. S3a, red arrows). DNA inside the bacterial biofilm was further quantified by detecting the fluorescence intensity of DNA images in the yellow areas (Figure 4b and Supplementary Fig. S3b). The DNA content inside biofilms formed by the *prsA*-deficient mutant was significantly reduced compared to wild-type and *prsA*-complemented strains (Figure 4b and Supplementary Fig. S3b). To confirm reduction of eDNA inside the vegetation, we extracted the total DNA of the vegetations without lysing the bacteria [7]. Semi-quantitative assays using 16S rRNA gene specific primers consistently showed that the bacterial eDNA levels in vegetation samples of the *prsA*-deficient mutant were significantly lower than that in samples of wild-type and the *prsA*-complemented strains (Figure 4(c-e), Supplementary Fig. S3c and S3d) [7]. These data suggested that PrsA mediates bacterial lysis or bacterial eDNA release that may contribute to biofilm formation in IE pathogenesis.

### PrsA contributes to the secretion and surface localization of AtlA

**Figure 5.**
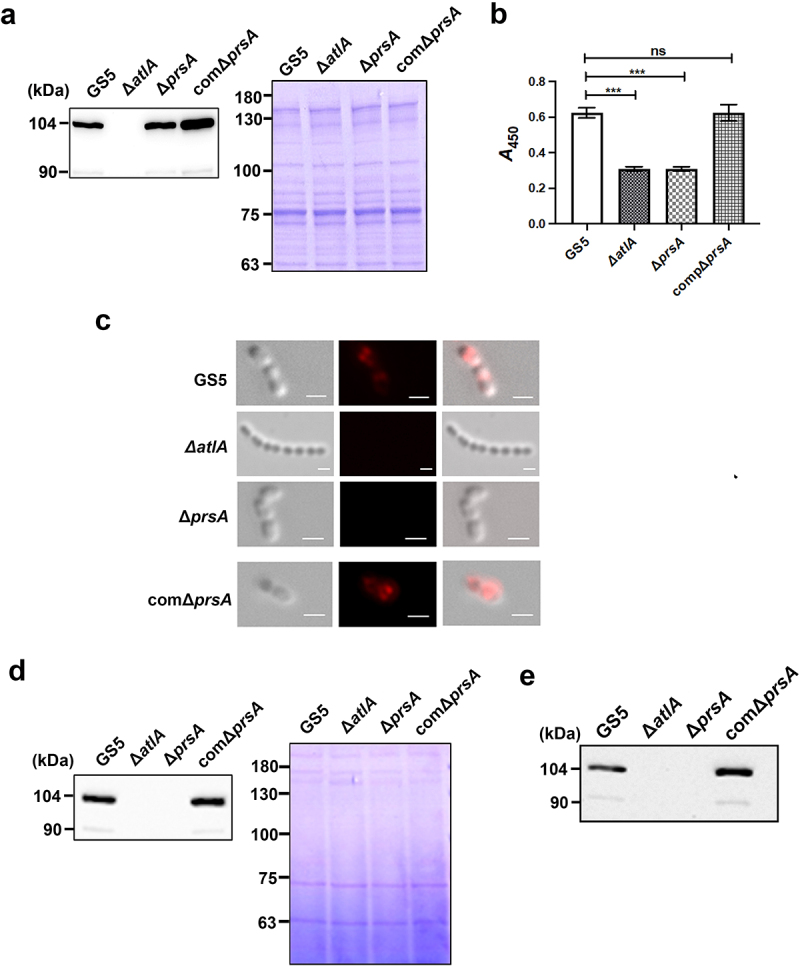
PrsA mediates surface exposure and secretion of AtlA. (a) Cell wall/envelope-associated proteins of *S. mutans* GS5 wild-type, Δ*prsA*, and comΔ*prsA* were extracted with 4% sodium dodecyl sulfate (SDS) sample buffer. AtlA was detected by western blotting. (b and c) AtlA exposure on the bacterial surface detected by whole-cell ELISA (b) and immunofluorescence staining using anti-AtlA antibodies (c). Data of whole-cell ELISA are presented as the means ± standard deviation of triplicate data, and were analyzed by 1-way ANOVA. ***P < 0.001, ns, not significant. The result is a representative experiment from three independent experiments. The results of two other repeats were available in Supplemental Materials. (b). Immunofluorescence staining of AtlA on the bacterial surface was detected by ZEISS Apotome 3D Super Resolution Microscopy (c). Bar indicates 1μm. (d) AtlA secretion to the culture medium detected by western blotting. Cell-free overnight culture supernatants from *S. mutans* wild-type, Δ*prsA*, and comΔ*prsA* were concentrated 20-fold, and analyzed by western blotting using anti-AtlA antibodies. (e) Cell wall pulldown of bacterial proteins in the culture supernatant of *S. mutans* wild-type, Δ*prsA*, and comΔ*prsA* detected by western blotting using anti-AtlA antibodies.

The characteristics of the *prsA*-deficient and *atlA*-deficient strains are similar in terms of enhanced cell chain length, reduced autolysis activity, reduced ability to release eDNA, and reduced biofilm forming capacity *in vitro* and *in vivo* [7,25]. Therefore, we investigated whether PrsA affects AtlA expression or function by extracting bacterial cell wall/membrane-associated proteins from total bacteria or biofilm populations with 4% SDS buffer and analysing the extracts by western blotting. The expression of cell wall/envelope-associated AtlA was similar for wild-type and mutant strains (Figure 5a, Supplementary Fig. S4a and S5a). However, in whole cell ELISA to detect cell surface exposure of bacterial proteins, AtlA exposure on the bacterial surface was reduced in the *prsA*-deficient mutant strain compared to the wild-type and *prsA*-complemented strains in both whole bacteria and biofilm populations (Figure 5b, Supplementary Fig. S4b and S5b). The result of immunofluorescence staining also showed that the reduced AtlA localization on bacterial surface of the *prsA*-deficient mutant strain (Figure 5c). In addition, the amount of secreted AtlA was dramatically reduced in the bacterial culture medium of the *prsA*-deficient mutant strain compared with the wild-type and *prsA*-complemented strains (Figure 5d, Supplementary Fig. S4c and S5c). A previous study indicated that AtlA binds to serotype c cell wall carbohydrates and mediates bacterial cell wall separation [13]. To further confirm that secreted AtlA can bind to bacterial surfaces, we performed a cell wall pulldown assay with bacterial culture medium supernatants [13] and used western blotting to examine the binding of secreted AtlA in the culture medium of wild-type or the *prsA*-complemented strain to bacterial cell walls (Figure 5e). Together, these data suggested that PrsA mediates secretion and surface localization of AtlA.

## Complementation of AtlA in the culture medium restored eDNA-dependent biofilm formation by the *prsA*-deficient mutant strain

**Figure 6.**
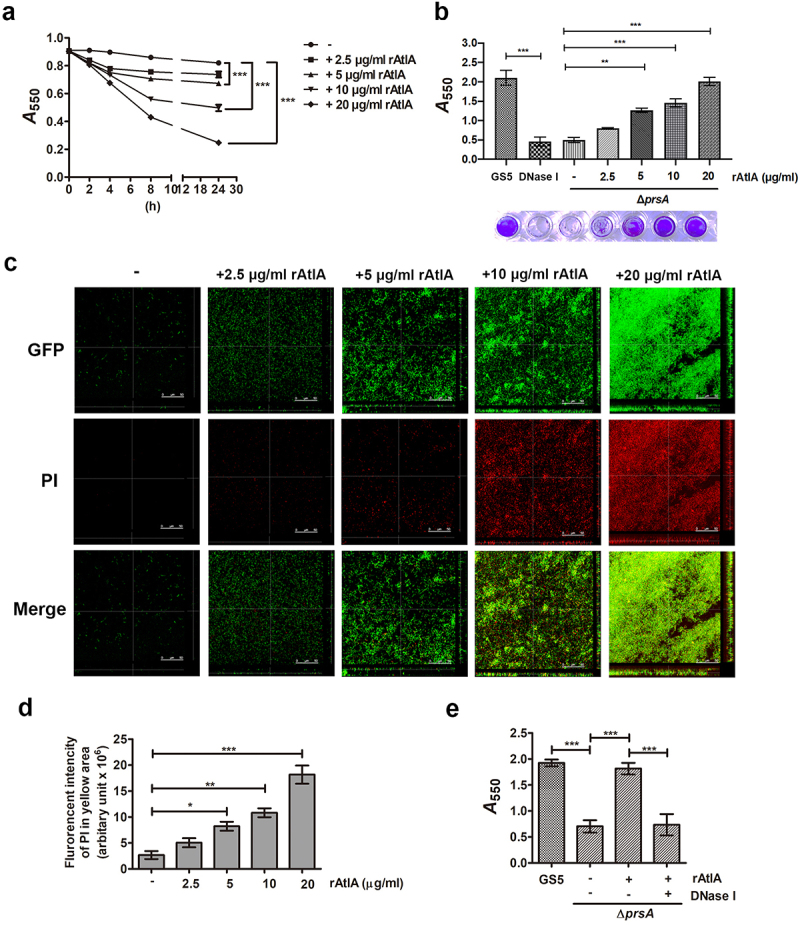
Addition of recombinant AtlA to the culture medium restores the capacity of the *prsA*-deficient mutant strain to form eDNA-dependent biofilm. (a) Bacterial autolysis assessed by measuring the OD_550_ of the cell suspension of the *prsA*-deficient mutant strain containing a concentration series of recombinant AtlA (rAtla). Data are expressed as the mean ± standard deviation of triplicate data; values at 24 h were analyzed by 1-way ANOVA, ***P < 0.001. (b) Quantification of biofilms of *prsA*-deficient mutant strain (Δ*prsA*) cultured in medium containing indicated concentrations of recombinant rAtla using a crystal violet staining assay. The experiment was performed in triplicate, and the data are presented as the mean ± standard deviation, and were analyzed by 1-way ANOVA. ***P < 0.001, ns, not significant. (c) Confocal laser scanning microscopy images of Δ*prsA* biofilms cultured in medium containing a series of rAtla concentrations (630× magnification). *S. mutans* GS5 wild-type and mutant strains were transformed with pPdgfpuv (green), and bacterial eDNA was stained with 10 μM propidium iodide (PI). GFP, green fluorescent protein; PI, propidium iodide. (d) Quantification of eDNA inside the biofilm by detecting the fluorescence intensity of propidium iodide staining. The quantified values of the extended focus images of biofilms were detected using ImageJ software, and statistically analyzed by 1-way ANOVA. The means ± standard deviation of three independent experiments is presented. ***P < 0.001, **P < 0.01, and *P < 0.05. (e) Quantification of *S. mutans* biofilm by a crystal violet assay. *S. mutans* GS5 wild-type and Δ*prsA* biofilms were grown in the culture medium with or without 20 μg/ml rAtla or 5 unit/ml DNase I. Means of OD_550_ absorbance readings ± standard deviation of triplicate data is shown; ***P < 0.001 by 1-way ANOVA. The results for a representative experiment from three independent experiments are shown. The results of two other repeats were available in Supplemental Materials.

**Figure 7.**
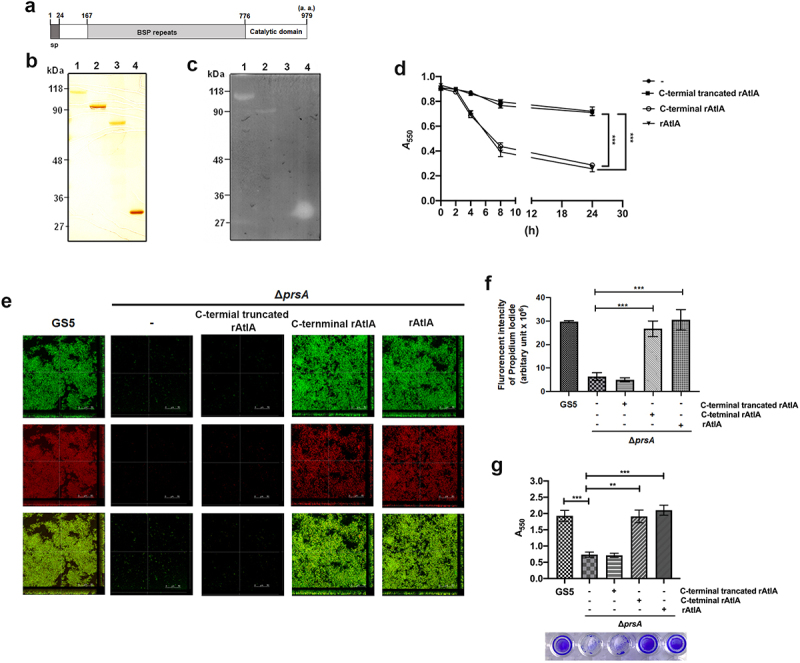
AtlA enzyme activity is important to PrsA-mediated bacterial autolysis and biofilm formation. (a) Schematic representation of the domain structure of AtlA. sp, signal peptide. (b) the virous recombinant AtlA proteins (rAtla) were separated by SDS-PAGE followed by silver staining. Lane1, full length of AtlA (residues 24–979); lane 2, mature form of AtlA (residues 167–979); 3, C-terminal truncation isoform (residues 167–776); lane 4, putative catalytic domain of AtlA (residues 776–979) (c) Zymographic analysis of rAtla enzyme activities. The rAtla proteins were separated by SDS-PAGE containing 1% (wet weight) heat-killed *S. mutans* cells. (d) Bacterial autolysis assessed by measuring the OD550 of the cell suspension of the *prsA*-deficient mutant strain containing 20 μg/ml indicated rAtla isoforms. The experiment was performed in triplicate and repeated three time. Data are expressed as the mean ± standard deviation of triplicate data. The values at 24 h were analyzed by 1-way ANOVA, ***P < 0.001. (e) Confocal laser scanning microscopy images of Δ*prsA* biofilms cultured in medium containing 20 μg/ml indicated rAtla isoforms (630× magnification). *S. mutans* GS5 wild-type and mutant strains were transformed with pPdgfpuv (green), and bacterial eDNA was stained with 10 μM propidium iodide (PI). GFP, green fluorescent protein; PI, propidium iodide. (f) Quantification of eDNA inside the biofilm by detecting the fluorescence intensity of propidium iodide staining. The quantified values of the extended focus images of biofilms were detected using ImageJ software, and statistically analyzed by 1-way ANOVA. The means ± standard deviation of three independent experiments is presented. ***P < 0.001. (g) Quantification of *S. mutans* biofilm by a crystal violet assay. *S. mutans* GS5 wild-type and Δ*prsA* biofilms were grown in the culture medium with or without 20 μg/ml indicated rAtla isoforms. Means of OD_550_ absorbance readings ± standard deviation of triplicate data are shown; ***P < 0.001, **P<0.01, by 1-way ANOVA. The results for a representative experiment from three independent experiments are shown. The results of two other repeats were available in Supplemental Materials.

To further explore the role of secreted AtlA in PrsA-mediated biofilm formation, the recombinant AtlA (rAtlA) was used [21]. Addition of rAtlA dose-dependently enhanced the autolysis activity of the *prsA*-deficient mutant strain (Figure 6a). Consistently, complementation with rAtlA in the culture medium dose-dependently restored eDNA release to the biofilm matrix as well as the capacity of the *prsA*-deficient mutant strain to form biofilms (Figure 6(b-d)). Treatment with DNase I reduced the rAtlA-enhanced biofilm formation of the *prsA*-deficient mutant strain, suggesting that addition of rAtlA in the culture bacterial medium indeed enhanced bacterial eDNA release that contributed to biofilm formation by the prsA-deficient mutant strain (Figure 6e). We next used the recombinant AtlA proteins having a C-terminal truncation (residues 167–776) or the putative AtlA C-terminal catalytic domain (residues 776–979) to understand whether AtlA enzyme activity was important for PrsA-mediated biofilm formation (Figure 7(a,b)) [7]. The autolysis activity of the recombinant full length and C-terminal domain of AtlA were confirmed using a zymogram assay (Figure 7c). Addition of recombinant full length or the C-terminal domain of AtlA restored the autolysis activity and eDNA-dependent biofilm formation of prsA-deficient mutant strain (Figure 7(d-g)). However, the C-terminal truncated mutant of AtlA had no effect. These data emphasized the important role of AtlA enzyme activity in PrsA-mediated biofilm formation and also confirmed the role of secreted AtlA in PrsA-mediated biofilm formation.

## Discussion

PrsA has a role in mediating virulence factor secretion and surface localization in many bacteria. Results of this study demonstrate for the first time that prsA mediates *S. mutans* virulence in a fatal disease, IE. Our previous studies demonstrated the important role of AtlA-mediated bacterial eDNA release in biofilm formation on damaged heart valves in IE [7]. AtlA was also shown to bind to bacterial cell wall carbohydrates to mediate bacterial autolysis and cell division [13]. Although a previous study indicated that PrsA also has a role in bacterial cell division, there was no direct evidence that indicated a relationship between PrsA and AtlA [20]. In the present study, we provide evidence for an important role for PrsA in surface localization and release of AtlA that contributes to eDNA release and biofilm formation.

Bacterial PPIases are divided into three superfamilies: FKBPs, cyclophilins, and parvulins [15]. PPIases such as PrsA modulate virulence in bacterial pathogens [15]. PrsA is an *N*-terminally lipid-anchored lipoprotein that belongs to the parvulin family [13,27]. Located in the compartment between the cell membrane and cell wall, PrsA is thought to chaperone folding of bacterial virulence proteins destined for cell export [13,27]. Bacterial proteins exported to the plasma membrane by the Sec translocase are unfolded [28]; thus, subsequent correct folding is critical for their secretion and function. PrsA located in the membrane–wall interface promotes appropriate protein folding by catalysing cis-trans isomerization of peptide bonds preceding prolyl residues [15,29]. In *Bacillus subtilis*, misfolded penicillin-binding proteins (PBP2a) were observed in the *prsA* mutant strain [30], and in *S. aureus* PrsA functions not only in the secretion of proteases and phospholipase, but also affects the enzyme activity of these secreted virulence factors [17]. Our data showed that in a Δ*prsA*-deficient mutant strain, AtlA retained its association with the bacteria cell wall/envelope, but had reduced autolysis activity (Figure 3c, 5a, Supplementary Fig. S2c and S5a). This data suggests an important role for PrsA in modulating the enzyme activity of AtlA on the bacterial cell wall. Our data also showed that PrsA mediates AtlA secretion (Figure 5, Supplementary Fig. S4 and S5), and a recent study indicated that AtlA specifically binds to serotype c carbohydrates in the bacterial cell wall to mediate bacterial cell division and autolysis [13]. Based on our findings and those that were previously described, we propose a hypothetical model in which PrsA located inside the cell wall regulates AtlA by chaperoning its folding and secretion (Supplementary Fig. S6). The secreted AtlA then sequentially binds to cell wall carbohydrates and mediates both cell autolysis and eDNA release that contributes to biofilm formation on damaged heart valves in IE.

Regarding *S. mutans* virulence, previous studies described several mutant strains that display similar phenotypes to the *prsA*-deficient mutant, including *pcp*-, *liaR*- or *ropA*-deficient mutant strains. These similarities include increased chain length and unchanged AtlA expression in the cell envelope/wall [8,20]. RopA is a bacterial PPIase that belongs to the FKBP family [15], whereas LiaR and PCP are two regulatory proteins that are also involved in eDNA release and biofilm formation in IE pathogenesis [8]. These earlier studies also showed that AtlA expression levels associated with the cell envelope were not changed in these isogenic mutant strains compared with the wild-type strain. Interestingly, our preliminary data indicated that AtlA secretion was reduced in the medium of *pcp*-, *liaR*- or *ropA*-deficient mutant strain cultures, but only the addition of rAtlA could restore the biofilm formation capacity of the *ropA*-deficient mutant strains (Supplementary Fig. S7). These data suggested that, similar to PrsA, *S. mutans* RopA may also play a role in the secretion of AtlA. RopA is a homolog of trigger factor, a ribosome-associated chaperone that is involved in the biogenesis and maturation of newly formed proteins [31] and has been shown to contribute to the maturation and secretion of the *S. pyogenes* cysteine protease SspB [32]. In *S. mutans*, inactivation of ropA reduced bacterial genetic competence as well as acid and peroxide tolerance, in addition to biofilm formation in the presence of saliva components [33]. Our preliminary data showed a similar phenotype for the *ropA*- and prsA-deficient mutant strains. We will further investigate the actions of PrsA and RopA in the secretion of AtlA in future studies. We also observed a reduction in the amount of AtlA in the culture medium for *liaR*- and *pcp*- deficient mutant strains, but the addition of AtlA could not restore the phenotype, suggesting that, in addition to mediating AtlA secretion, LiaR and PCP may also influence AtlA function or affect bacterial autolysis machinery. Future studies should be performed to address how LiaR and PCP could affect AtlA function or bacterial autolysis machinery in IE pathogenesis.

In addition to IE, *S. mutans* is known to form biofilms on dental surfaces that are mediated by glucan synthesis catalysed by the glucosyltransferase (Gtf) system [34,35]. Previous studies also showed that eDNA crosslinks glucan to stabilize *S. mutans* biofilms on dental surfaces [36] and that glucosyltransferase-I may also be involved in eDNA-dependent biofilm formation *in vitro* [37]. Although reduced expression of glucosyltransferases and glucan production was also seen for the *prsA*-deficient mutans strain [14], our data showed that addition of eDNA or rAtlA restored biofilm-forming capacity of the *prsA*-deficient *S. mutans* stain (Figure 3i and 6b), indicating an important role for eDNA and AtlA in *prsA*-mediated biofilm formation. In contrast to dental surfaces, there is no sucrose in plasma, and the glucosyltransferase-deficient strain GHS1DD, which showed reduced ability to form glucan-dependent biofilms relative to GS5 (Supplementary Fig. 8), had no reduction in the capacity to cause IE [24]. This finding suggested that Gtfs and Gtf-mediated glucans may play limited roles in *S. mutans* (at least in GS5)-induced IE. However, we cannot exclude the role for glucan in other *S. mutans* strains or oral streptococci-induced IE. Indeed, previous studies indicated that bacterial binding to exopolysaccharides is important for immune evasion, as well as for the capacity to colonize heart valves in IE [38,39]. In addition, before entering circulation, oral streptococci may be enmeshed in sucrose-derived exopolysaccharides. Therefore, the role of PrsA-mediated glucan in the pathogenesis of IE requires further investigation.

The mechanism of biofilm formation on the heart valve *in vivo* is complex and involves bacterial immune evasion as well as adherence to endothelial cells and extracellular matrix proteins. Therefore, in addition to influencing AtlA secretion, PrsA may also affect the secretion or surface-localization of other bacterial virulence proteins that are involved in the pathogenesis of *S. mutans*-induced IE, and these putative virulence proteins require further investigation.

In conclusion, the results obtained in the present study demonstrated the important role of PrsA-mediated virulence in the pathogenesis of IE and that PrsA modulates the secretion and surface localization of AtlA to promote eDNA-dependent biofilm formation, which contributes, at least in part, to PrsA-mediated IE pathogenesis. Since autolysin-mediated eDNA-dependent biofilms are also observed in other species [11,40,41], and several bacterial species carry PrsA homologues, the results obtained in this study could provide important information not only about the pathogenesis of *S. mutans*-induced IE, but could also enhance understanding of mechanisms associated with other infectious diseases that involve biofilm formation.

## Supplementary Material

Supplemental Material

## Data Availability

The authors confirm that the data supporting the findings of this study are available within the article and its supplementary materials.
