# Supplementary material for: Streptococcus mutans PrsA mediates AtlA secretion contributing to extracellular DNA release and biofilm formation in the pathogenesis of infective endocarditis
Source: Virulence. 2022 Aug 11;13(1):1379–92. doi: 10.1080/21505594.2022.2105351 (PMC9377233; doi:10.1080/21505594.2022.2105351)
Supplement: Supplemental Material [file KVIR_A_2105351_SM3219.zip › supplementary/Supplementary Figures.pdf]

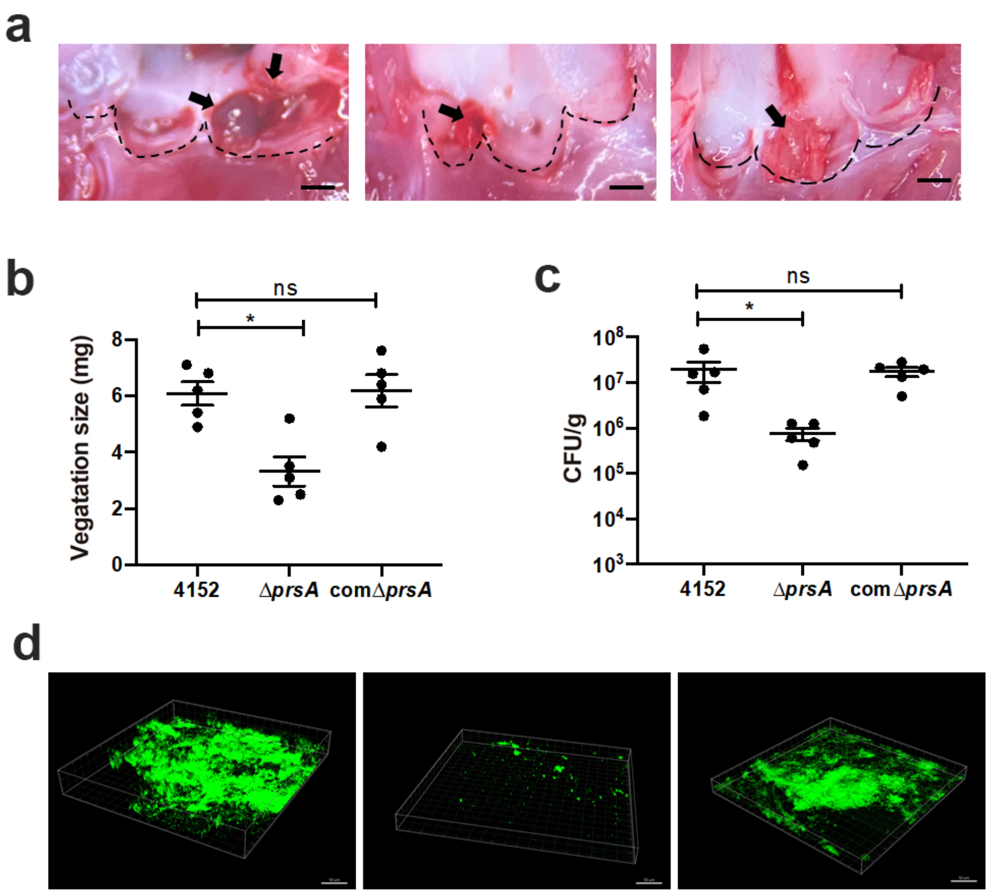

Supplementary Figure 1 *PrsA*-deficient mutant strain shows reduced ability to promote vegetation formation in a rat model of IE in rats. Investigation of the role of PrsA in the pathogenesis of IE using wild-type, Δ*prsA,* and comΔ*prsA* strains of a clinical isolate 4152 in rat models of endocarditis. (a) Vegetation formation on the heart valves of endocarditis rats. Markers represent vegetation (black arrows) and valves (dashed lines). Scale bars represent 1 mm. (b and c) The number of colonized bacteria inside vegetation (b) and vegetation size (c) was measured. Data are presented as a scatter plot with mean ± standard error of the mean. *$P < 0.05$ by Kruskal-Wallis test with subsequent Dunn's test; ns, not significant. (d) Three-dimensional structure of GFP-tagged *S. mutans* biofilms inside the vegetation that was harvested from injured heart valves and observed by

confocal laser scanning microscopy (630× magnification). Bars indicate 50 μm.

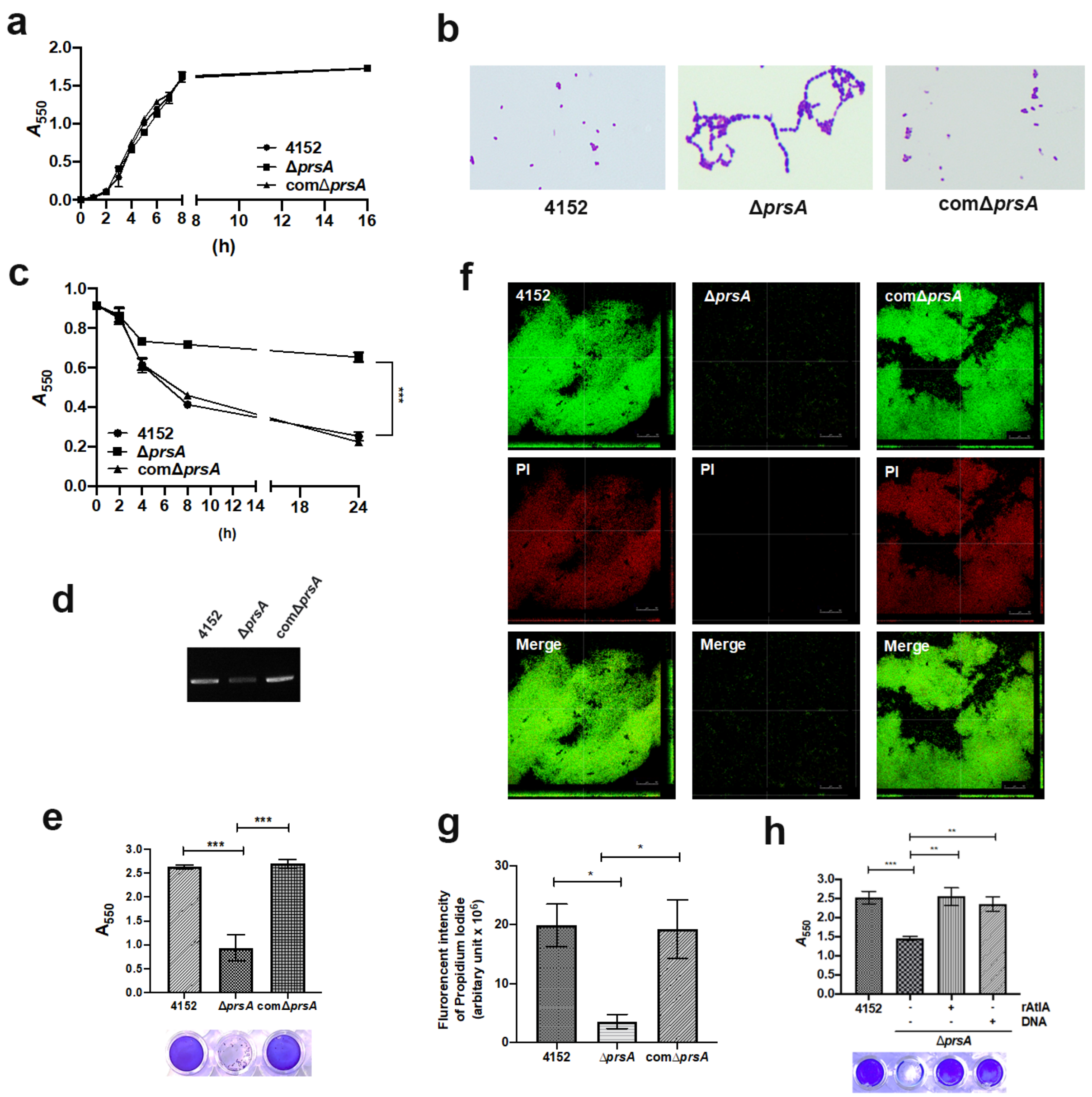

Supplementary Figure 2 PrsA mediates *S. mutans* cell separation, autolysis, eDNA release, and eDNA-dependent biofilm formation. (a) The bacterial growth was measured by detecting the absorbance of cultures at 550 nm. (b) Appearance of wild-type, Δ*prsA*, and comΔ*prsA* strains of *S. mutans* clinical blood isolate 4152 in the stationary phase were observed by light microscopy. (c) Bacterial autolysis assessed by measuring the OD_{550} of the cell suspensions. Data are expressed as the mean ± standard deviation of triplicate data. The values at 24 h were analyzed by 1-way ANOVA,

***P < 0.001. (d) Semi-quantitative analysis of bacterial eDNA release by PCR. PCR products obtained with 16S rRNA primers were analyzed on 1% agarose gels. (e) Quantification of *S. mutans* biofilm formation using a crystal violet staining assay. Means of OD_{550} absorbance readings ± standard deviation of triplicate data are shown, and the data were statistically analyzed by 1-way ANOVA. **P < 0.01, ns, not significant. (f) Confocal laser scanning microscopy images of *S. mutans* biofilms (630× magnification). *S. mutans* GS5 wild-type and mutant strains were transformed with pPDGFPuv (green), and bacterial eDNA was stained with 10 μM propidium iodide (PI). GFP, green fluorescent protein. (g) Quantification of eDNA inside biofilms by detecting the fluorescence intensity of propidium iodide staining. The quantified values of the extended focus images of biofilms were determined using ImageJ software. The data were analyzed by 1-way ANOVA from three independent experiments and are presented as the mean value ± standard deviation. ***P < 0.001 by. ns, not significant. (h) Quantification of biofilm by crystal violet staining. *S. mutans* blood isolate 4152 wild-type or Δ*prsA* strains were grown in culture medium with or without 10 μg/ml bacterial DNA or 20 μg/ml rAtlA. The data are presented as the mean ± standard deviation, and were analyzed by 1-way ANOVA. **P < 0.01, ***P < 0.001. The results for a representative experiment from three independent experiments are shown.

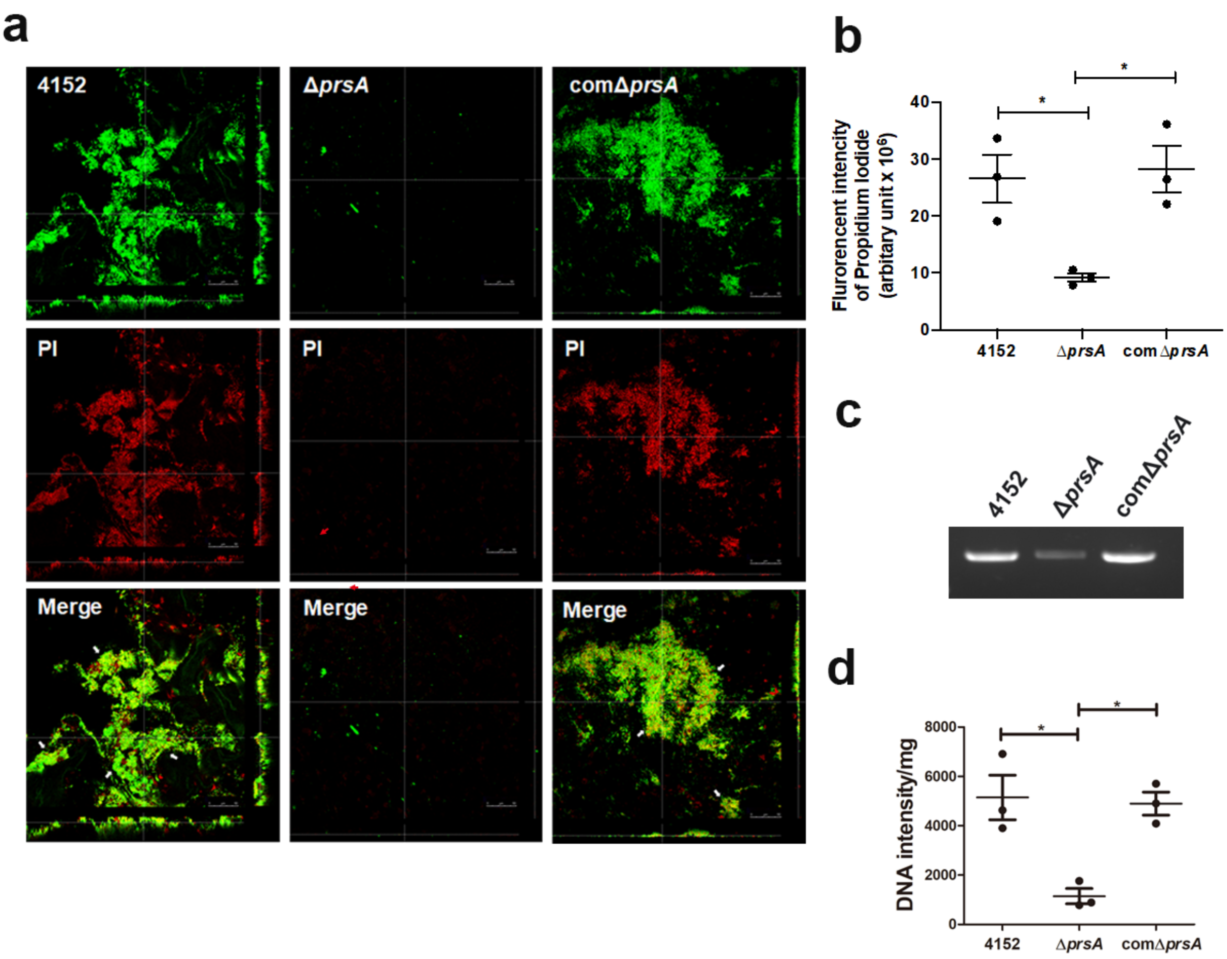

Supplementary Figure 3 PrsA mediates *in vivo* eDNA-dependent biofilm formation. (a) Confocal laser scanning microscopic images of biofilm formation inside the vegetation (630× magnification). Wild-type and mutant strains of *S. mutans* clinical blood isolate 4152 were transformed with pPDGFPuv (green), and bacterial eDNA was stained with 10 μM propidium iodide (PI). GFP, green fluorescent protein. The red arrows indicate small bacterial aggregates without eDNA inside, and white arrows indicate the presence of both *S. mutans* and eDNA (yellow areas). The images shown are representative of three independent experiments. (b) Quantification of the eDNA inside the bacterial biofilms (yellow areas) by measuring the fluorescence intensity of propidium iodide staining. The quantified values of the extended focus images of biofilms were detected using ImageJ software. The

data are presented as scatter plots with mean ± standard deviation. *$P < 0.05$ by 1-way ANOVA. (c and d) Semi-quantification of bacterial eDNA inside the vegetation. Total DNA of vegetation was extracted without lysing bacteria, and the bacterial 16S rRNA gene was amplified by PCR with specific primers. PCR products were analyzed on 1% agarose gels (c) and the intensity was semi-quantified using ImageJ software (d). The data were statistically analyzed by 1-way ANOVA and presented as scatter plots with means ± standard deviation from three independent experiments, *$P < 0.05$.

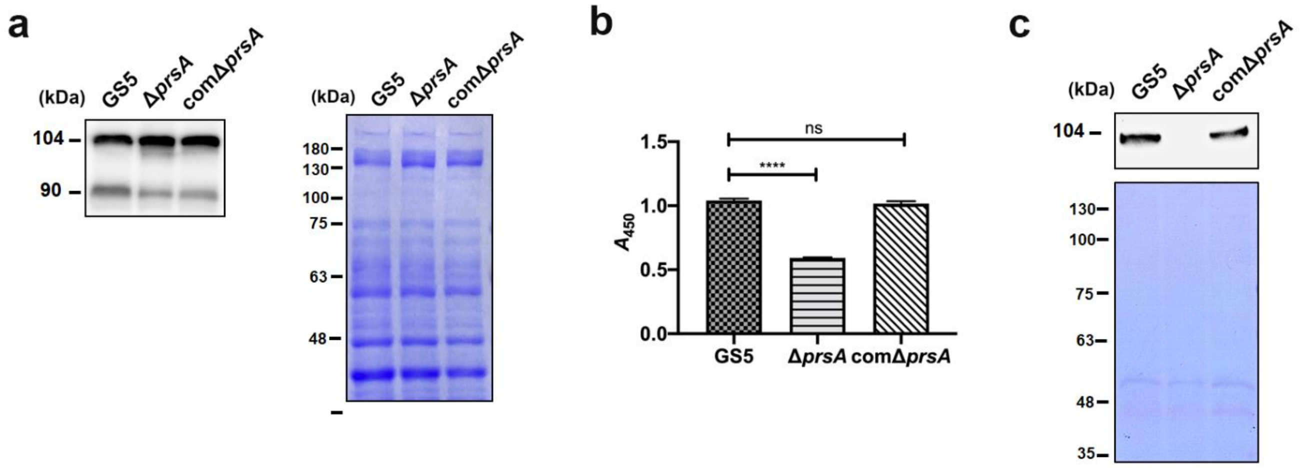

Supplementary Figure 4 PrsA mediates surface exposure and secretion of AtlA in biofilm populations of *S. mutans* GS5 (a) Cell wall/envelope-associated proteins of the biofilm populations of *S. mutans* GS5 wild-type, Δ*prsA*, and comΔ*prsA* were extracted with 4% sodium dodecyl sulfate (SDS) sample buffer. AtlA was detected by western blotting. (b) AtlA exposure on the bacterial surface detected by whole-cell ELISA using anti-AtlA antibodies. Data are presented as the means ± standard deviation of triplicate data and were analyzed by 1-way ANOVA. ****P* < 0.001, ns, not significant. (c) AtlA secretion to the culture medium detected by western blotting. Cell-free supernatants from *S. mutans* wild-type, Δ*prsA*, and comΔ*prsA* biofilm cultures were concentrated 20-fold, and analyzed by western blotting using anti-AtlA antibodies. The results are a representative experiment from three independent experiments.

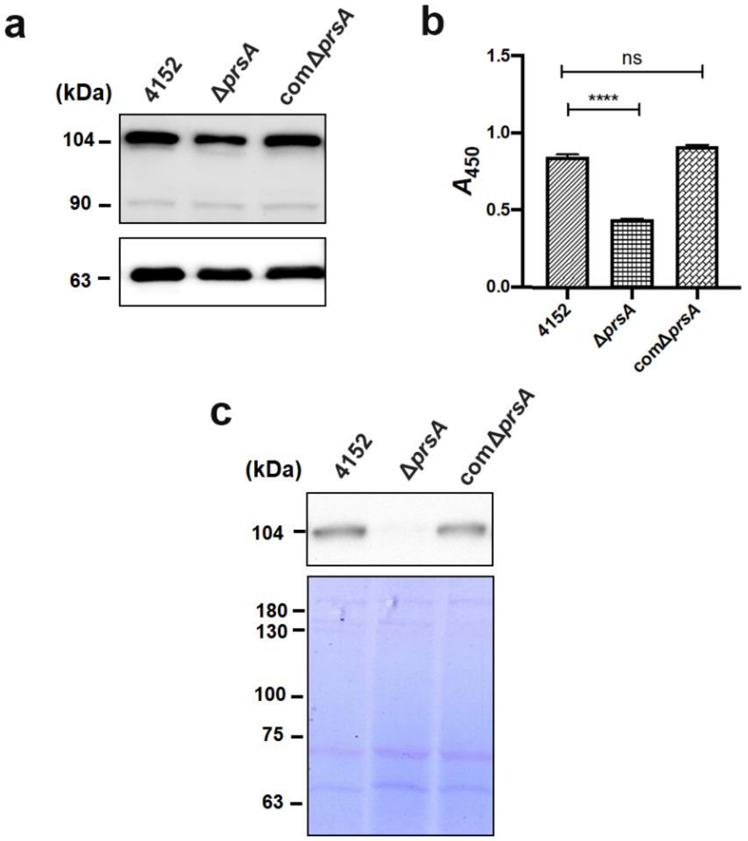

Supplementary Figure 5 PrsA mediates surface exposure and secretion of AtlA. (a) Cell wall/envelope-associated proteins of wild-type, Δ*prsA*, and comΔ*prsA* of clinical blood isolate 4152 were extracted with 4% sodium dodecyl sulfate (SDS) sample buffer. AtlA (upper panel) and GbpB (bottom panel, as loading control) were detected by western blotting. (b) AtlA exposure on the bacterial surface detected by whole-cell ELISA using anti-AtlA antibodies. Data are presented as the means ± standard deviation of triplicate data and were analyzed by 1-way ANOVA. ***$P < 0.001$, ns, not significant. (c) AtlA secretion to the culture medium detected by western blotting. Cell-free overnight culture supernatants from 4152 wild-type, Δ*prsA*, and comΔ*prsA* were concentrated 20-fold, and analyzed by western blotting using anti-AtlA antibodies. The results are a representative experiment from three independent experiments.

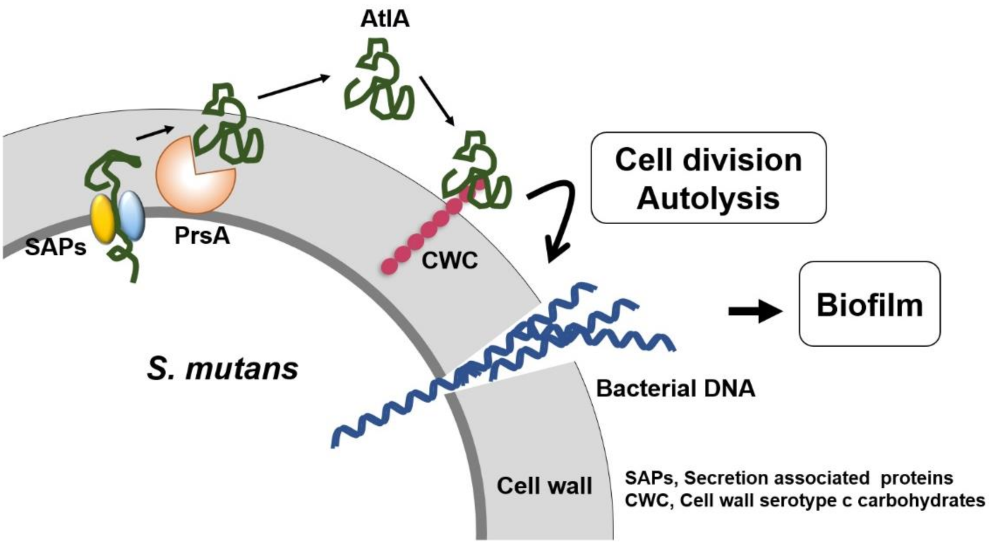

Supplementary Figure 6 The hypothetical model for PrsA-mediated AtlA release that contributes to bacterial cell division, autolysis, and biofilm formation. PrsA located inside the cell wall contributes to folding and secretion of AtlA. AtlA sequentially binds to cell wall carbohydrates and mediates cell autolysis and eDNA release, which contributes to biofilm formation on damaged heart valves in IE.

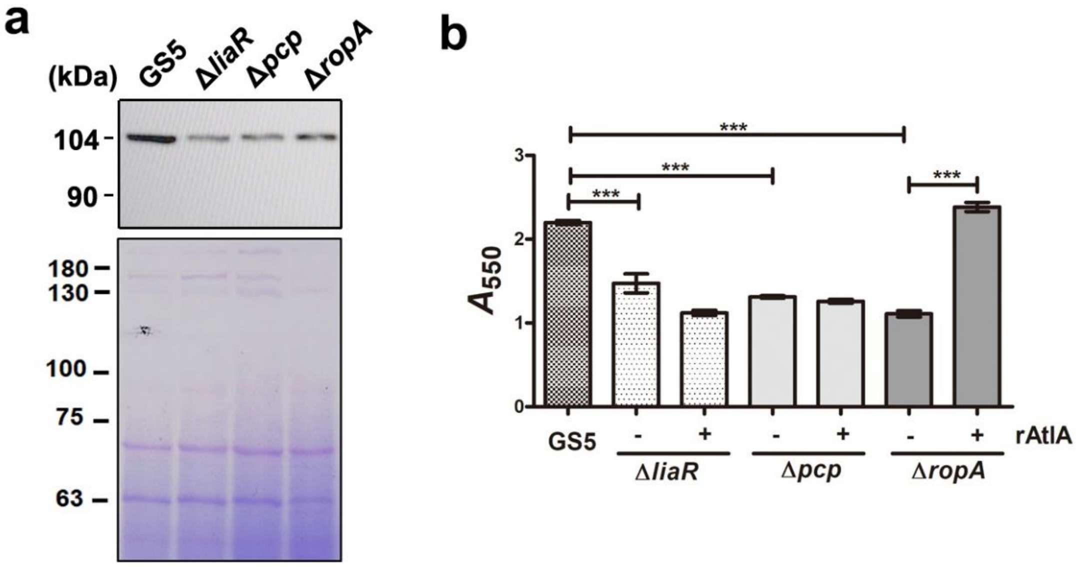

Supplementary Figure 7 RopA mediates AtlA secretion, contributing to biofilm formation. (a) AtlA secretion to the culture medium detected by western blotting. Cell-free overnight culture supernatants from *S. mutans* wild type, Δ*liaR*, Δ*pcp* and Δ*ropA* were concentrated 20-fold, separated by 7.5% SDS-PAGE, and stained with Coomassie blue (bottom panel) and analyzed by western blotting using anti-AtlA antibodies (upper panel). (b) Biofilms of *S. mutans* GS5 wild type, Δ*liaR*, Δ*pcp* and Δ*ropA* grown with or without 20 μg/ml rAtlA that were stained with 0.1% crystal violet and quantified by measuring the absorbance at 550 nm. Data are expressed as the mean ± standard deviation; ****P* < 0.001 by 1-way analysis of variance. These experiments were repeated three times and results for a representative experiment are shown.

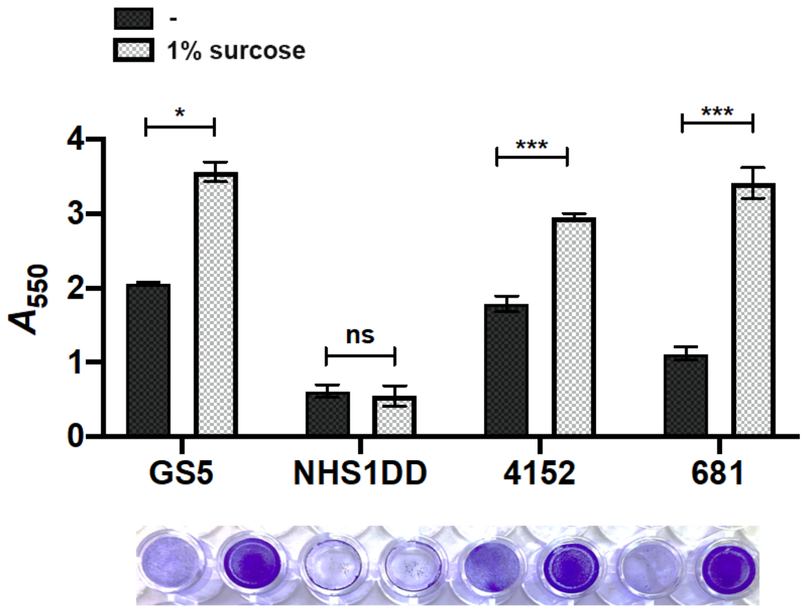

Supplementary Figure 8 ***S. mutans*** **GS5 and 4152 have the ability to form the glucan-dependent biofilm.** *S. mutans* GS5 wild type and *gtfs*-deficient mutant (HHS1DD) strains, and clinical blood isolates 4152 and 681 were culture in BHI medium with or without containing 1% sucrose and the biofilms were stained with 0.1% crystal violet and quantified by measuring the absorbance at 550 nm. Data are expressed as the mean ± standard deviation; ***P < 0.001, *P < 0.05 by 1-way analysis of variance. The experiment was repeated three times and results for a representative experiment are shown.
