# Supplementary material for: Streptococcus mutans PrsA mediates AtlA secretion contributing to extracellular DNA release and biofilm formation in the pathogenesis of infective endocarditis
Source: Virulence. 2022 Aug 11;13(1):1379–92. doi: 10.1080/21505594.2022.2105351 (PMC9377233; doi:10.1080/21505594.2022.2105351)
Supplement: Supplemental Material [file KVIR_A_2105351_SM3219.zip › supplementary/Supplementary Materials.pdf]

Results of two other independent experiments for the figures depicting graphs

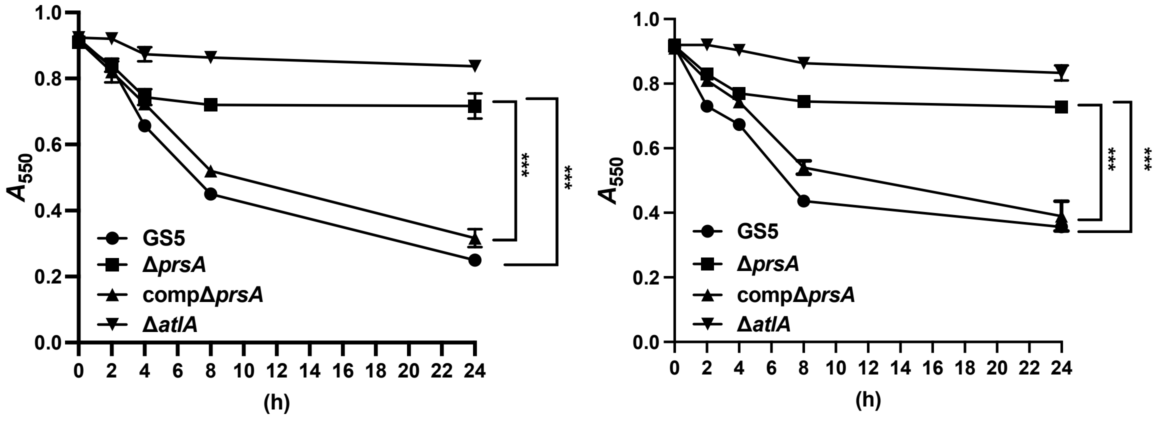

Result of two other independent experiments of Figure 3c.

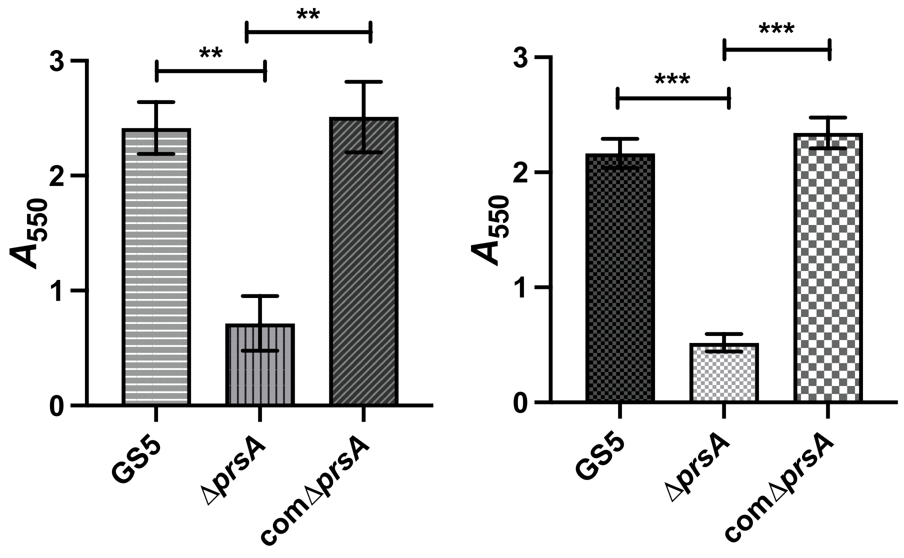

Result of two other independent experiments of Figure 3f.

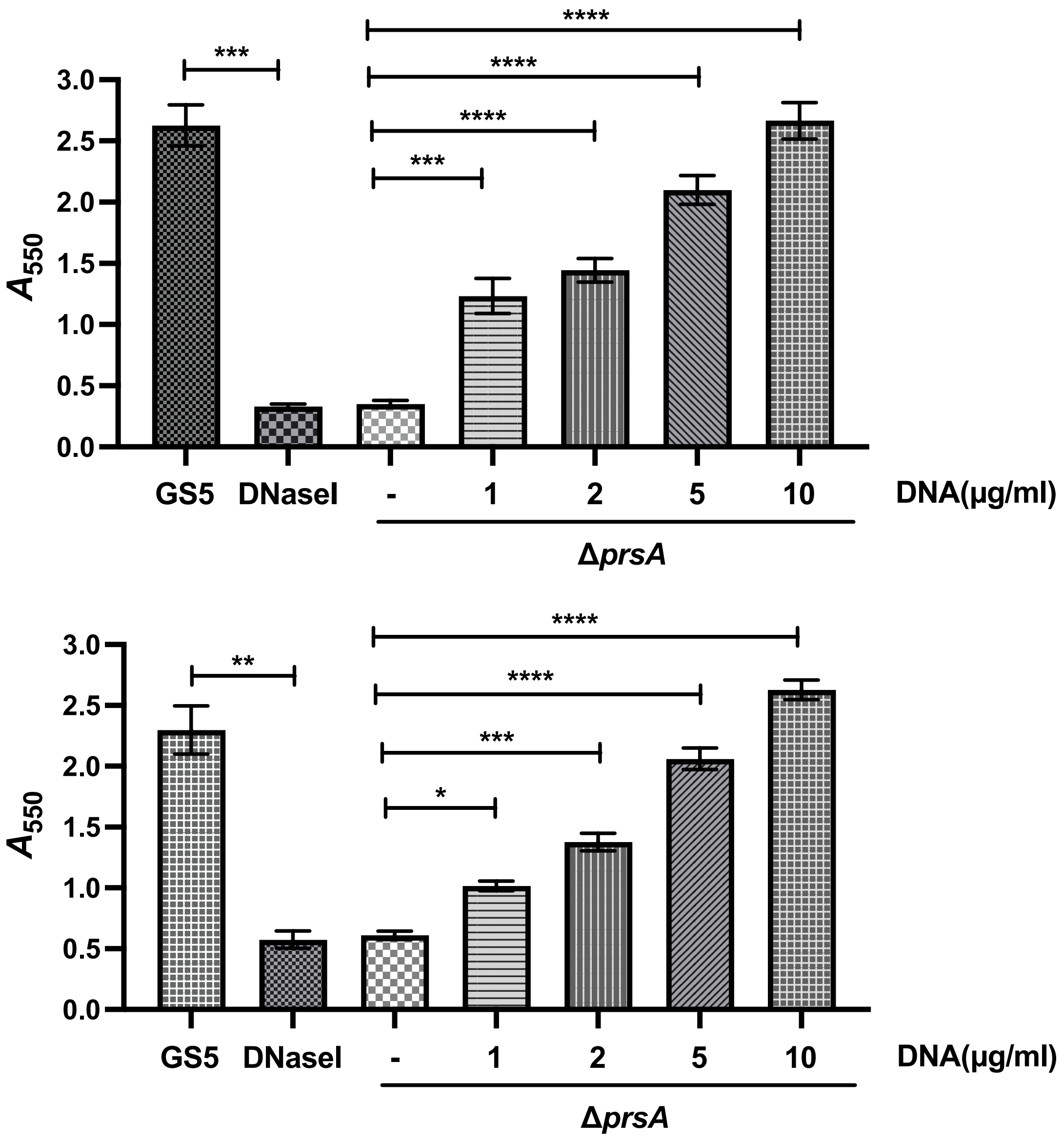

Result of two other independent experiments of Figure 3i.

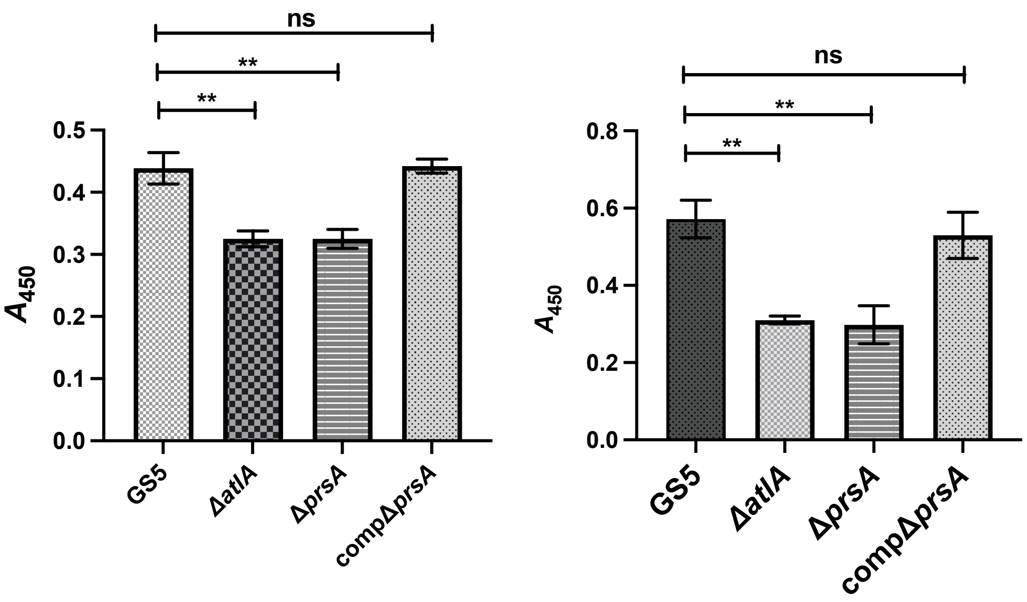

Result of two other independent experiments of Figure 5b.

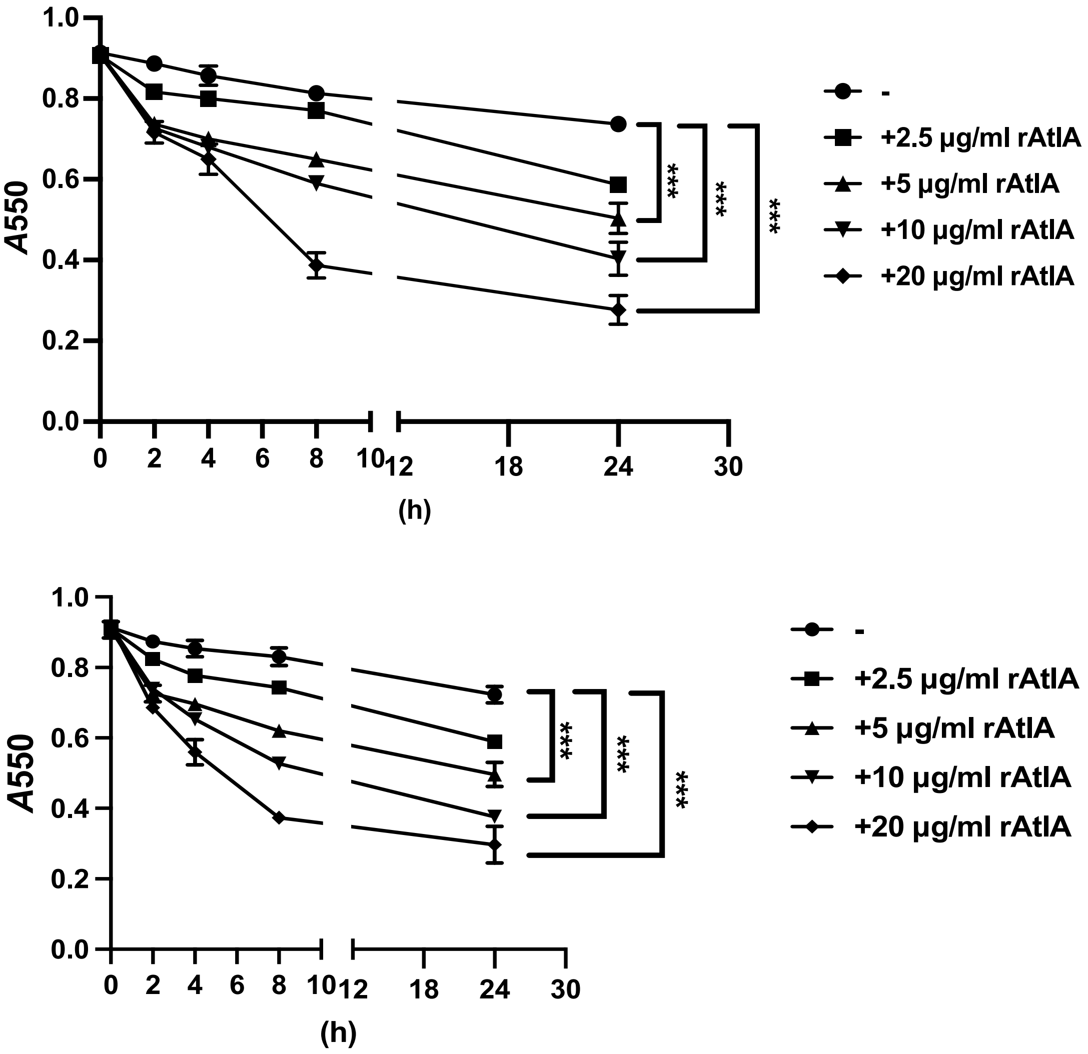

Result of two other independent experiments of Figure 6a.

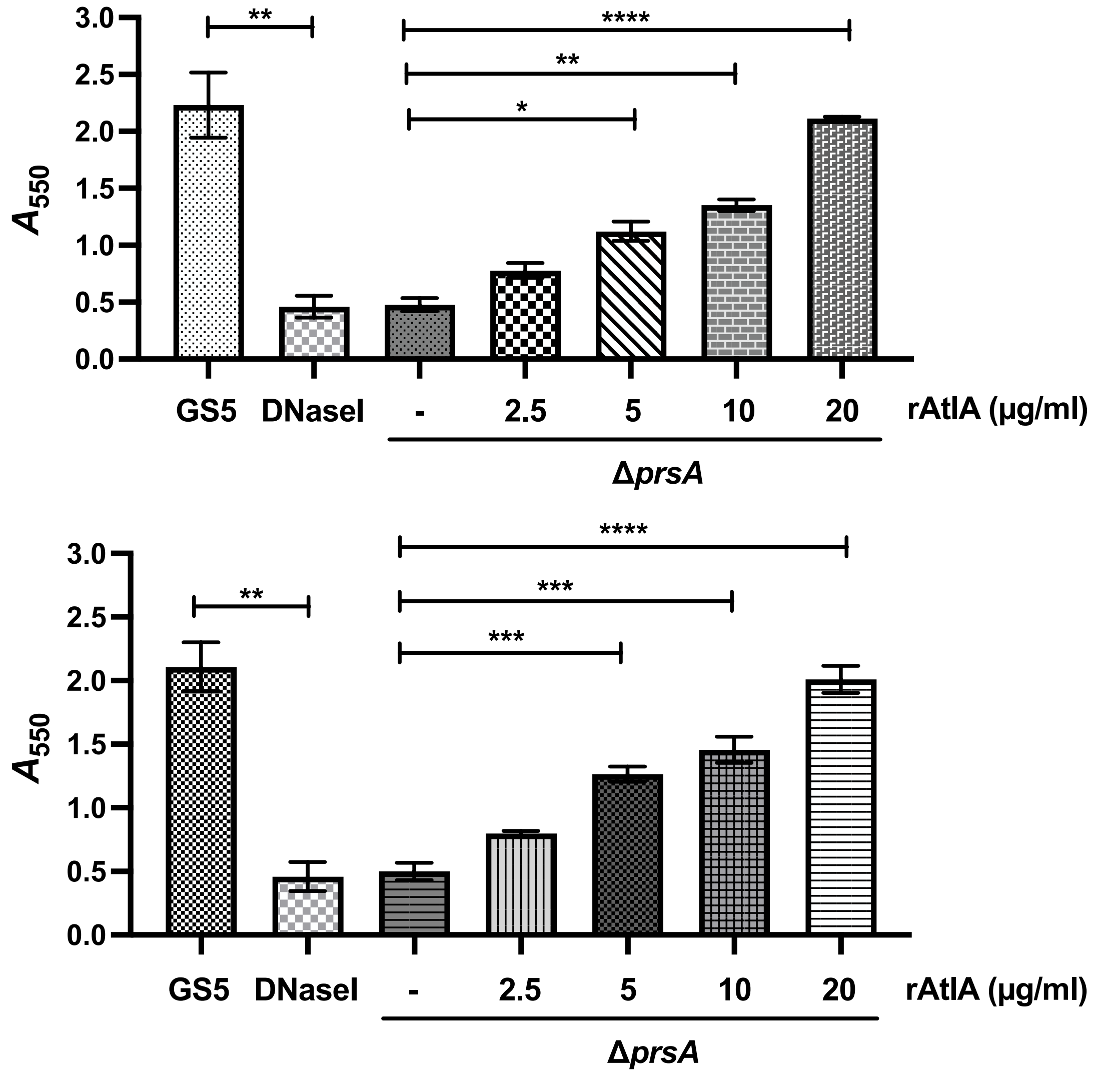

Result of two other independent experiments of Figure 6b.

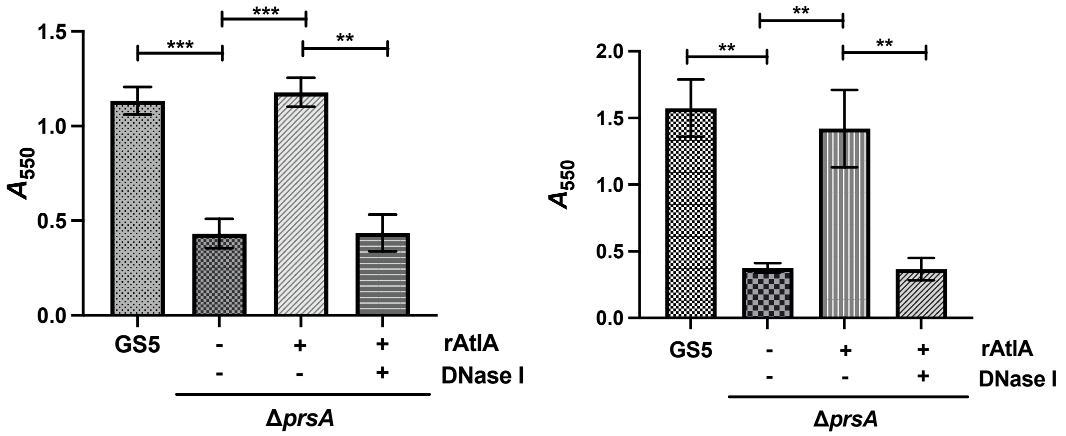

Result of two other independent experiments of Figure 6e.

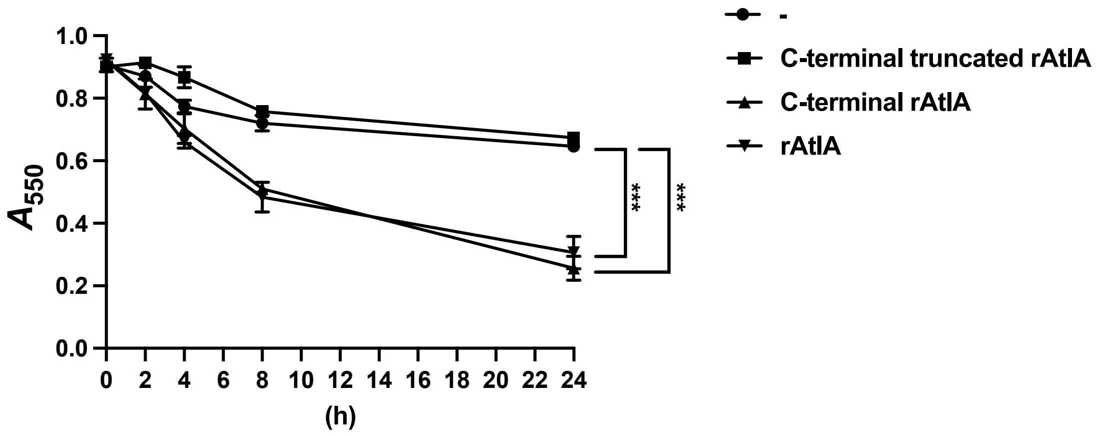

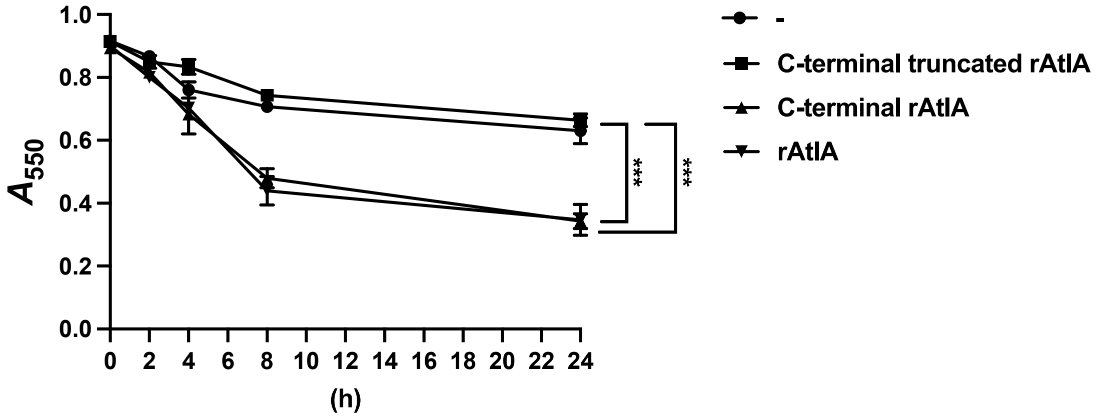

Result of two other independent experiments of Figure 7d.

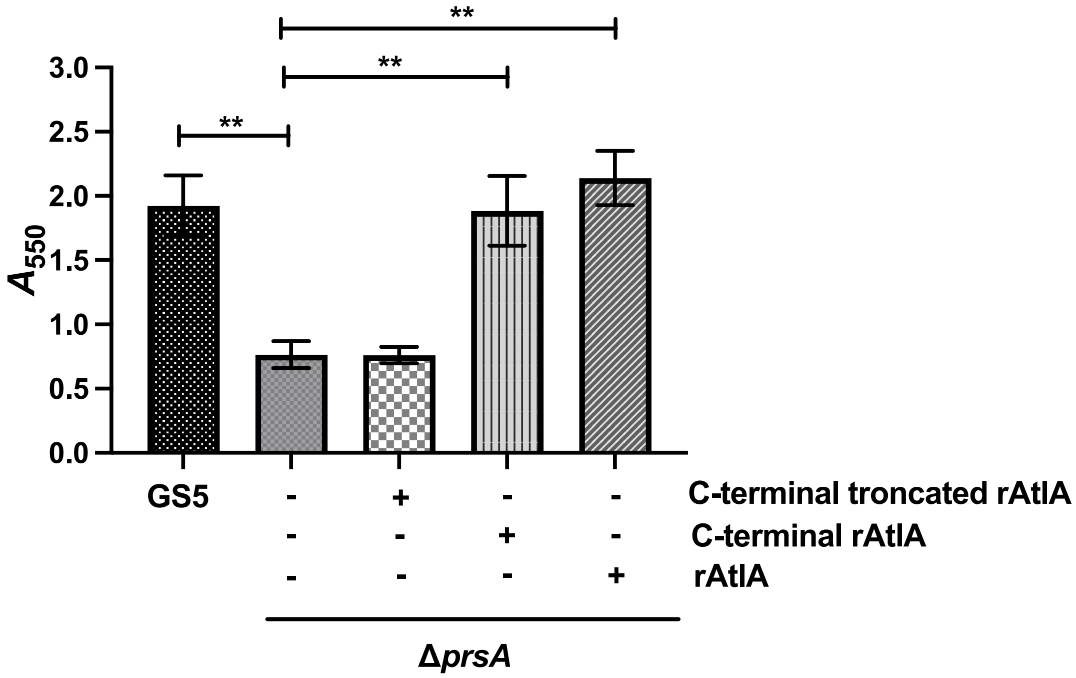

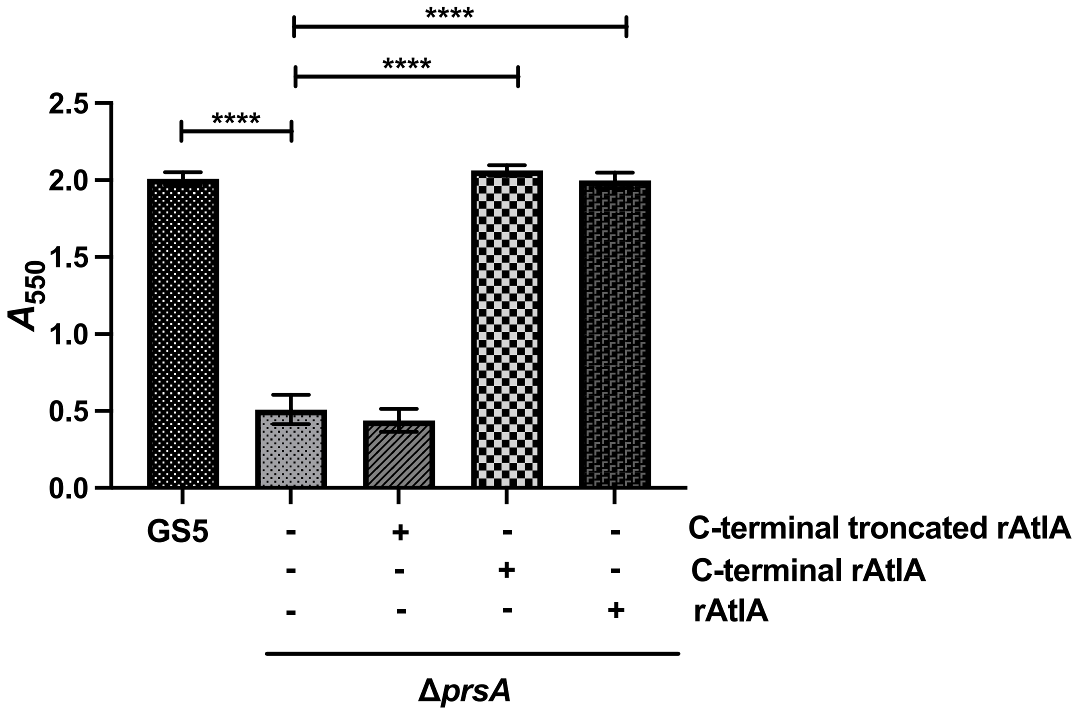

Result of two other independent experiments of Figure 7g.

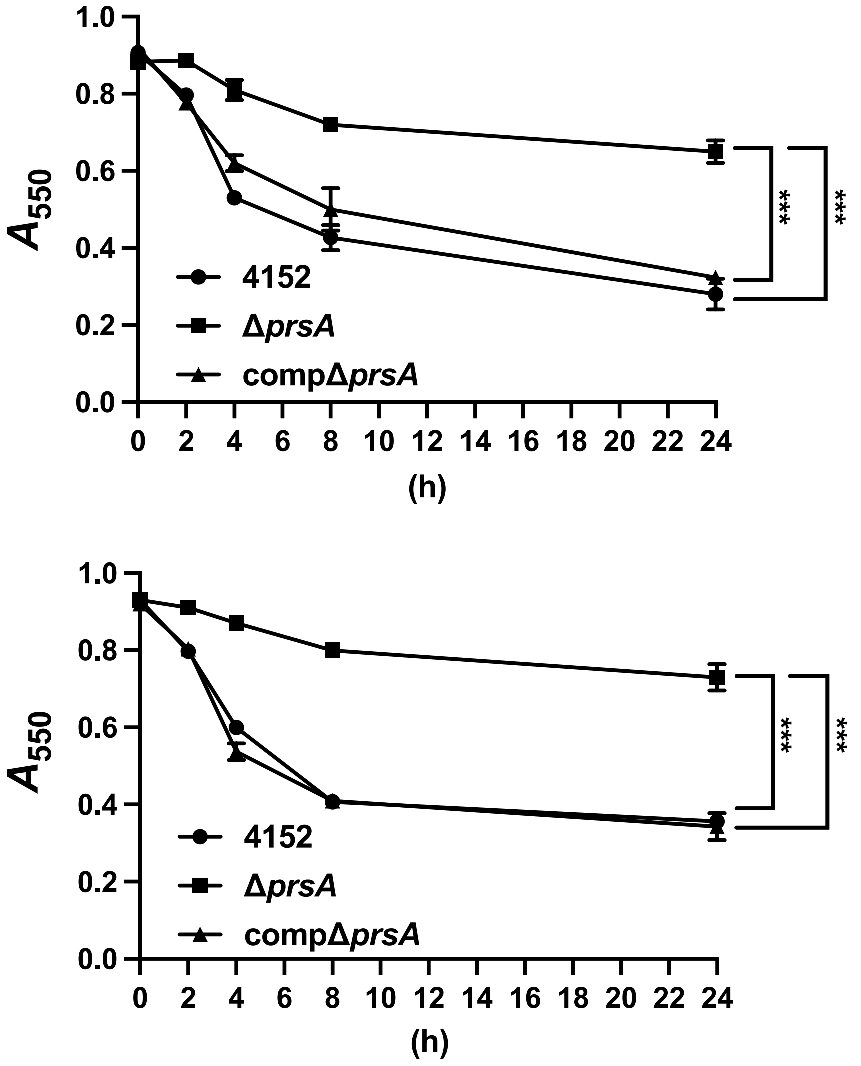

Result of two other independent experiments of supplementary Figure 2c.

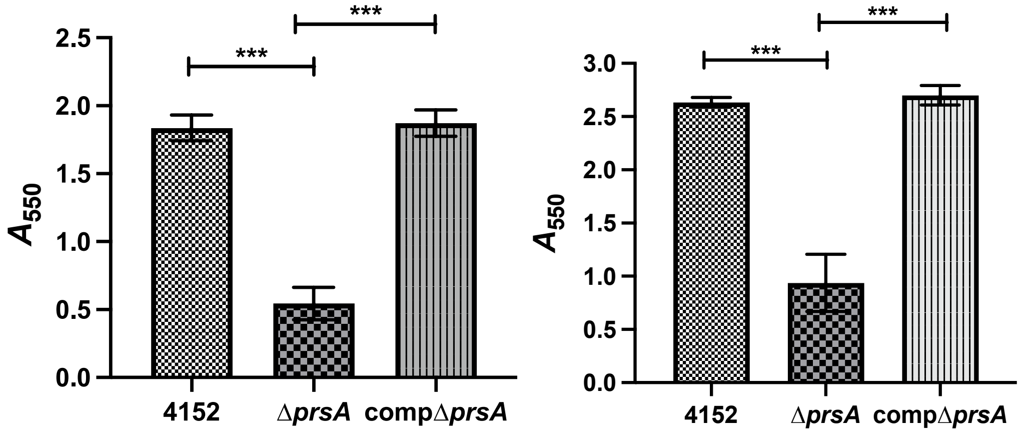

Result of two other independent experiments of supplementary Figure 2e.

.

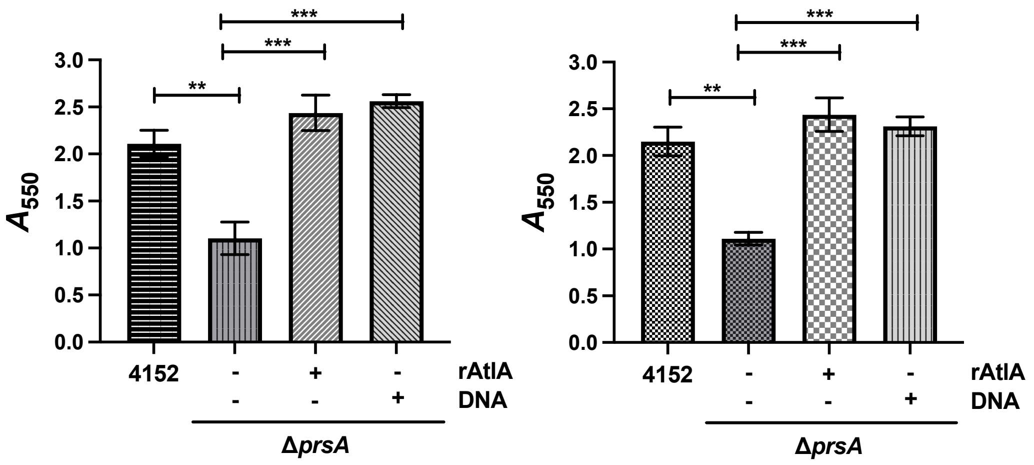

Result of two other independent experiments of supplementary Figure 2h.

.

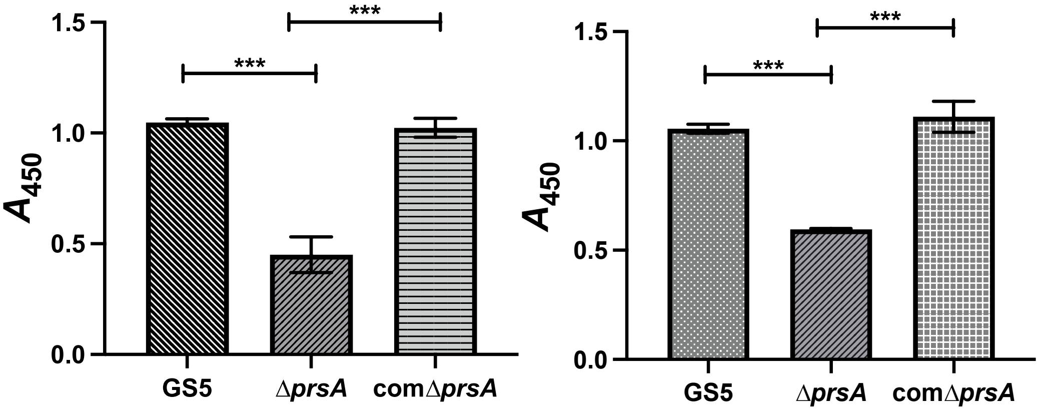

Result of two other independent experiments of supplementary Figure 4b.

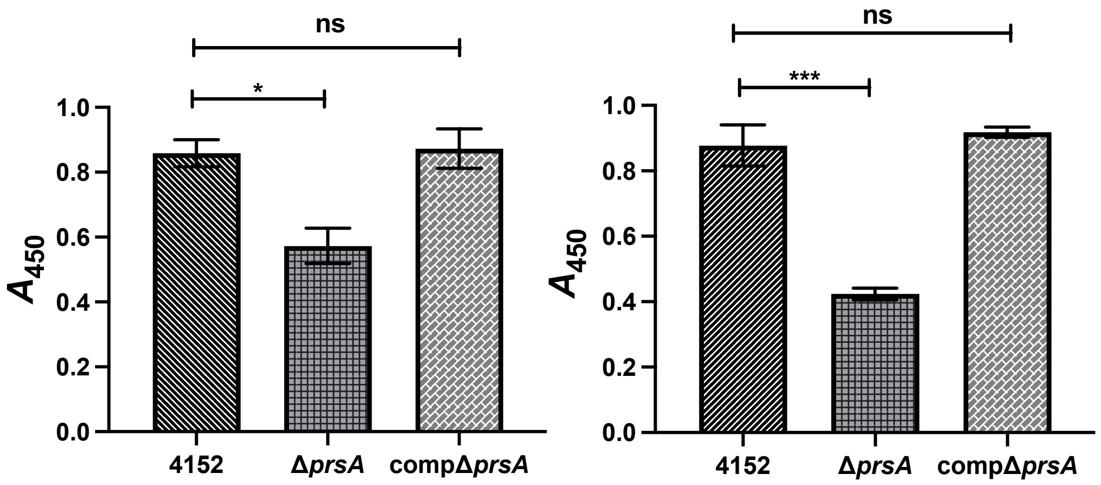

Result of two other independent experiments of supplementary Figure 5b.
